# Collaboration Between People Admitted to Acute Mental Health Units, Their Family Members and Nurses in the Detection of Mental State Changes and Recovery: A Qualitative Systematic Review

**DOI:** 10.1111/inm.70244

**Published:** 2026-04-24

**Authors:** Karen Foster, Linda Coventry, Yvonne Middlewick, Lindsay Smith, Beverley Ewens

**Affiliations:** ^1^ School of Nursing and Midwifery, Edith Cowan University Joondalup Western Australia Australia; ^2^ Centre for Nursing Research Sir Charles Gairdner Hospital Nedlands Western Australia Australia; ^3^ School of Nursing, Paramedicine, & Healthcare Sciences Charles Sturt University Bathurst New South Wales Australia; ^4^ School of Nursing University of Tasmania Launceston Tasmania Australia; ^5^ School of Nursing College of Health and Education, Murdoch University Murdoch Western Australia Australia

**Keywords:** collaboration, family, inpatient, lived experience, mental illness, nurse, patient

## Abstract

Mental health nurses can engage with people with mental health challenges and their families due to their presence on acute mental health units. This presence helps to facilitate recovery‐focused care within a physically and emotionally safe environment. Mental health care should be person‐centred, individualised, and include wherever possible family and other people in the person's support network. The objective of this systematic review was to explore the existing qualitative literature that describes experiences of collaboration between people with lived experience of mental health challenges, their family members, and nurses in relation to changes in mental state during an admission to an acute mental health unit. Qualitative systematic review. The Joanna Briggs Institute methodology for systematic reviews of qualitative evidence was followed, including the JBI quality appraisal checklist. The databases searched were CINAHL, Medline, Scopus, Emcare and PsycInfo. Included articles were written in English and published between January 2013 and September 2023. Data was extracted that provided the perspectives of adults (18–64 years), their family members and nurses (collectively known as the “Triangle of Care”) regarding communication when mental state changes during an acute mental health admission to a public hospital. Perspectives of the three groups were explored separately then synthesised. The ConQual approach was used to assess confidence in the findings. This review included articles on the lived experience perspective (*n* = 17), the family perspective (*n* = 1), and the nurse perspective (*n* = 13). The identified articles presented common perspectives on how to facilitate person‐first nursing care that is supportive of personal recovery, including the provision of one‐to‐one discussions and inviting input from family members. Nurses, in particular, highlighted systemic barriers and facilitators to the focus on personal recovery in clinical practice. Communication between the three members of the “Triangle of Care” is essential for the identification of mental state changes as a critical aspect of providing nursing care that promotes personal recovery. All three members of the “Triangle of Care” highlighted the importance of making time for one‐on‐one conversations, which are impacted by other factors such as competing demands on time and confidentiality. Further research is indicated to improve clinical practice and service provision, with extant research being particularly sparse in non‐Western and non‐English speaking regions.

**Literature Review Protocol:** Prospero registration number: CRD420250639443

## 1 Introduction

### 1.1 The Global Perspective

It is estimated that there are one billion people worldwide living with a diagnosable mental health disorder (World Health Organization 2022). However, the provision of specialised mental health clinicians and treatment approaches remain inadequate, particularly in developing countries (World Health Organization 2021). Global objectives for the provision of mental health care include establishing and maintaining effective leadership and governance as well as the implementation of strategies for promotion and prevention at all stages of the life span, including suicide prevention and ensuring the specific needs of the older population are met (World Health Organization 2021). These objectives however, rely on several principles to be successful, including enhancing the empowerment and autonomy of people with mental health challenges and psychosocial disabilities, and providing support and information to people experiencing those challenges and their families (World Health Organization 2021). Empowerment of people with lived experience of mental health services is reliant on individual's rights being upheld, including the provision of informed consent which is free of coercive practices such as forced treatment (World Health Organization and United Nations Human Rights Office of the High Commissioner 2023).

The rights of people with mental health challenges and their families to be involved in decisions about their own treatment is also protected in the Convention on the Rights of Persons with Disabilities (United Nations 2008). This gives rise to an obligation to replace a substituted decision‐making regime with a supported decision‐making regime (United Nations 2014). For example, family members should be engaged in the development of policy and ongoing service provision and also have their own support needs recognised and met (World Health Organization 2021). Despite these expectations, the response of mental health services and the inclusion of family members in mental health care remain inadequate (Eckardt 2022; World Health Organization 2022). Landeweer et al. (2017) argued this inadequate response may result from inadequate discussion of the differing perspectives and agendas which provide barriers for involving families of people with mental health challenges in collaborating with health staff.

### 1.2 Moving From the Biomedical Approach to a Personal Recovery Framework

The term “recovery” has been defined in various ways within the literature. Best et al. (2020) referred to two aspects of recovery in and from psychosis that are both outcome‐focused measures. The first is a symptomatic definition, focused on sustained remission of symptoms. Traditional psychiatric approaches have predominantly been shaped by the biomedical model (Wand 2013). This is a medically directed and linear approach that focuses on rapid symptom stabilisation and behaviour management, diagnosis and treatment (Watson et al. 2014; Waldemar et al. 2016). The definition of “clinical recovery” does not change between individuals, is rated by the clinician and makes assumptions about normality (Slade and Wallace 2017; Slade 2009). Assessments are focused on pathology (Wand 2015) and success is measured by symptom reduction and stabilisation (Hristodoulidis et al. 2022).

The second outcome‐focused definition is functional recovery, which focuses on successful integration to the community (Best et al. 2020). The focus of functional recovery is to establish capacity for independence and connection in those with lived experience, in conjunction with the management of ongoing acute symptoms that may initially lead to admission to a high acuity mental health setting (Davidson 2020). Reliance on a biomedical model can also reflect a culture that is risk averse and adopts a deficit‐based approach to risk assessment, management, accountability and containment (Callaghan and Grundy 2018). The practice of medicine occurs within institutions that maintain power through the knowledge held by medical doctors (Foucault 2004). This approach often potentiates the inherent power differentials in favour of staff and the development of hierarchies within teams, with doctors having the ultimate decision‐making power over people with lived experience of mental health challenges (Barnes et al. 2022; Crowe 2022; Solomon et al. 2021). The doctor, as described by Foucault (1989), uses their objective clinical gaze to select or filter out elements of the clinical presentation that align within a biomedical paradigm.

Mental health nursing is emerging from a reliance on the biomedical model with a focus on *outcomes* to the *process* of personal recovery. Originating in the service user movement, as opposed to professionals, personal recovery has been described as a “deeply personal, unique process of changing one's attitudes, values, goals, skills and/or roles” (Anthony 1993, 527). Personal recovery is a “journey into life, not an outcome to be arrived at” that promotes hope, choice, empowerment, use of support and information (Slade 2009, 38, Deegan 1996). This is encapsulated by CHIME which comprises connectedness, hope and optimism about the future, identity, meaning in life and empowerment (Leamy et al. 2011). The concept of personal recovery is originally a Western concept that more recently has been adopted within Asia (Kuek et al. 2023) and other societies that may be considered collectivist including the Middle East (Hawsawi et al. 2024). Emphasis on different aspects of CHIME may also differ across cultures; for instance, some people may have an increased importance on social connection and shared responsibility or have different interpretations of empowerment to others (Panadevo et al. 2025).

Personal recovery requires the development of a deeper therapeutic relationship between the person with lived experience and staff and focuses on individualised care that meets the goals, needs and priorities of the individual, including wherever possible the involvement and needs of family members who form the person's network of support (Waldemar et al. 2016; Francis 2014; Australian Government 2010). Personal recovery is promoted by the development of a therapeutic alliance between the person with mental health challenges, their families and staff members defined by the Carers Trust (2013) as the “Triangle of Care”. An emphasis on personal recovery both necessitates the shift to shared decision‐making whilst recognising the importance of the psychosocial elements of disability and the interaction between the person's external environment and their needs (Cooney et al. 2021). This includes education of the multidisciplinary team in shared decision‐making to enable the development of a culture that facilitates personal recovery and shared decision‐making (Newton‐Howes and Gordon 2022).

A philosophy of personal recovery aligns with a strengths‐based nursing model that values uniqueness, collaboration, self‐determination and a holistic approach to care. Strengths‐based nursing emphasises and utilises the strengths of individuals, family and the team to manage challenges, promote healing and evidence‐based clinical practice (Gottlieb et al. 2012; Gottlieb and Gottlieb 2017). Strengths‐based nursing requires leadership that is conducive to promoting the autonomy and empowerment of others, all within the context of the Triangle of Care (Gottlieb et al. 2021; Lavoie‐Tremblay et al. 2024).

People who are acutely unwell are usually admitted to mental health inpatient units when their symptoms are especially acute and/or they pose safety risks to themselves or others, often involuntarily using the relevant Mental Health legislation. A reduction and elimination of restrictive practices is important to promote safety and wellbeing, however, there is more to be done to positively impact the experience of individuals and their families in acute mental health settings (Hallett et al. 2025). Adverse experiences within these settings result from an interplay of the ecosystem i.e., the physical environment and systems such as processes and transition periods, including periods of deteriorating mental state or progress towards discharge, and impact of adverse experience on an individual's autonomy, control and choice (Hallett et al. 2025).

### 1.3 Safety Within Mental Health Settings and Recognition of Mental State Changes

Any health care setting presents a risk of harm to the person admitted, staff or visitors. In order to ameliorate risk and improve the quality and safety of care, health care organisations have published standards to guide clinicians. These include The Canadian Quality & Patient Safety Framework for Health Services (2020), the National Institute for Health Care Excellence (NICE) Quality Standards (National Institute for Health Care Excellence n.d.) and the Australian Standards of Care for Mental Health Services (Australian Government 2010) as well as National Standards that incorporate the recognition of clinical deterioration and escalation of care (Australian Commission on Safety and Quality in Health Care 2017b).

The nonlinear nature of recovery reflects periods of growth as well as setbacks (Substance Abuse and Mental Health Services Administration 2005), which may result in a fluctuating mental state during an inpatient admission, which in turn may increase the level of risk. In comparison to physical health deterioration, which can be measured objectively by vital sign measurements, it is impossible to measure mental state changes in the same manner (Lamont et al. 2024). Identification of mental state deterioration is reliant upon the skills, experience, resources, and collaborative history, which includes discussion of early warning signs of deterioration (Australian Commission on Safety and Quality in Health Care 2017a). The Australian National Standards require the involvement of people in their own care (Action 8.3, p 64) and establish processes for people with lived experience and their families to express concerns and escalate care (Action 8.7, p. 65). However, better recognition is required that families are key partners in the provision of mental health care (Carers Trust 2013) and are included in the identification and management of mental state changes Australian Commission on Safety and Quality in Health Care (2017b).

Priority must be placed on the early identification and management of mental health challenges and physical health that can lead to mental state deterioration during an acute mental health admission (Australian Commission on Safety and Quality in Health Care 2017a). The cause of mental state changes and their impact on mental health recovery may also have associated psychosocial factors such as financial deprivation, social isolation, or social exclusion. There is therefore a pressing clinical need to explore the perspectives of, and provide support to, people with lived experience, their family members, and nurses to identify and manage mental state deterioration and mental health recovery. Mental health challenges and substance misuse can co‐occur, with the concept of recovery from substance abuse being similar to personal recovery in the mental health context—personalised care that is directed by their goals (Brophy et al. 2023). Hove et al. (2023) also encouraged nurses to take a prominent role in developing and providing integrated nursing care for these comorbid conditions.

Therefore, the objective of this systematic review was to explore the experiences of people with lived experience of mental health challenges, their families, and mental health nurses in order to develop a shared understanding of changes in mental state in the acute inpatient context. Specifically, the review questions are:

1. What is the experience of people with lived experience of mental health challenges in developing a shared understanding of changes in mental state?
2. What is the experience of families that have a member with mental health challenges in developing a shared understanding of changes in mental state?
3. What is the experience of mental health nurses in developing a shared understanding of changes in mental state?

### 1.4 Definitions

This paper utilises the contemporary and person‐focused term “person with lived experience of mental health challenges” to encapsulate the ongoing nature of recovery unless directly quoting from a particular source. “Mental health challenges” will be used in preference to “mental illness” as it infers a more holistic and recovery‐focused perspective.

“Family” is defined by the person who is experiencing mental health challenges (Foster et al. 2016) and can include the person's spouse, parent, sibling, or friend (Wyder and Bland 2014). This means that a family member may not necessarily be related by blood, nor live with the person who lived with mental health challenges. The term “family” or “families and friends” is more inclusive than “carer” as it broadens the focus from caregiving to the relational aspects of support and recovery (Wyder, Bland, et al. 2018). This is also an inclusive definition, as it is recognised that “family” may have cultural significance, for example, within the Australian First Nations (Molloy et al. 2019; McGough et al. 2018), as well as for the gender diverse community (Martin et al. 2019).

## 2 Methods

A systematic review of qualitative literature was conducted that adhered to the Joanna Briggs Institute (JBI) methodology (Porritt et al. 2024) for systematic reviews of qualitative research. This review has been reported using the Preferred Reporting Items for Systematic Reviews and Meta‐analyses statement (Page et al. 2021) and the protocol was registered with JBI on 05/02/2025 and Prospero: Registration number CRD420250639443.

### 2.1 Search Strategy

A systematic electronic database search was conducted from 2013 to September 2023 for all English language articles in five databases, including Cumulative Index of Nursing and Allied Health Literature (CINAHL), Emcare, Scopus, Medline, and PsycInfo. Published literature was obtained from 2013 onwards to coincide with the introduction of the National Framework for Recovery‐Focused Practice within Australia (Australian Health Ministers' Advisory Council 2013). Search terms incorporated MeSH headings and keywords in three categories: population of interest (P), concept (C), and context (C). Boolean operators “OR” and “AND” were used to expand or restrict the search results, respectively. To further ensure comprehensive coverage, the reference lists of all included studies were searched to identify additional articles not captured with the original search. Additionally, a citation search was conducted using Web of Science and Google Scholar for all included articles. The complete search strategies are provided in Appendix S1.

### 2.2 Selection Criteria

Inclusion criteria comprised adults (18–64 years), persons with lived experience of mental health challenges; family members of a person with lived experience of mental health challenges; nurses with experience of caring for a person with lived experience of mental health challenges within the context of a public acute mental health inpatient admission. The design of studies was either qualitative or mixed methods where the qualitative data could be extracted.

Exclusion criteria included community or forensic nursing, any mental health service where visits were undertaken in homes, admission to mother‐baby units, the child and adolescent context, older person's mental health, drug and alcohol units, eating disorder units, and private mental health admission. Conference abstracts, grey literature and pre‐print articles were also excluded.

### 2.3 Study Selection

All retrieved articles were uploaded to Covidence (covidence.org), for effective management of the literature. Three separate literature searches were undertaken which focused on the person with lived experience of mental health challenges, family members and nurses. Each of the three Covidence projects followed the same literature management and search methods and screening. Two independent reviewers completed screening initially by title and abstract then full text, with a third person resolving cases of disagreement. Appendix S2 describes articles excluded after full text review with reasons.

### 2.4 Quality Appraisal

Quality appraisal of each selected article was undertaken using the 10 questions from the JBI Critical Appraisal Checklist for Qualitative Research (Lockwood et al. 2015). Two reviewers independently appraised each article, with a third person to resolve any discrepancies. In accordance with the JBI Guidelines, the ConQual approach (Munn et al. 2014) was used to establish confidence in the synthesised findings. Confidence is determined by assessing the dependability and credibility of the individual findings in the located studies.

Dependability was described as determining if the chosen method is suitable for the research question and the alignment between the methodology and method (Munn et al. 2014).

Five questions from the checklist are particularly relevant to determining dependability:

1. Is there congruity between the research methodology and the research question or objectives?
2. Is there congruity between the research methodology and the methods used to collect data?
3. Is there congruity between the research methodology and the representation and analysis of data?
4. Is there a statement locating the researcher culturally or theoretically?
5. Is the influence of the researcher on the research, and vice‐versa, addressed?

All qualitative studies initially begin with a ConQual rating of high. Downgrading for dependability is determined as follows: If there were 4–5 ‘Yes’ responses, dependability remained at current level; for 2–3 ‘Yes’ responses, dependability moved down 1 level (from high to moderate); for 0–1 ‘Yes’ responses, dependability moved down 2 levels (from high to low or moderate to very low). The dependability of the synthesised finding may then be downgraded based on an aggregate from the included findings.

Credibility of the included articles evaluated the ‘fit’ between the author's interpretation and the original data (Tobin and Begley 2004). This was assessed by comparing the conclusions drawn by the authors and the illustrations provided by original data. This ‘fit’ was then assessed as unequivocal, that is, illustration beyond reasonable doubt (U); equivocal, illustration lacking clear association (E); or unsupported, no illustration (US) (Munn et al. 2014).

Downgrading for credibility occurred when there were a mix of equivocal and unequivocal; the level decreases by one; all equivocal, the level decreases by two; or a mix of equivocal and unsupported by three and all unsupported by four. A summary of findings table is then used to include the significant elements of the review and how the ConQual Score was determined.

#### Summary of Findings

**Table:** 

Collaboration between people admitted to acute mental health units, their family members and nurses in the detection of mental state changes and recovery					
Synthesised finding	Type of research	Dependability	Credibility	ConQual score	Comments
Living experience perspective of collaboration between themselves, their family and nurses regarding mental state changes					
The experience of a mental health admission	Qualitative	High (no change)	Moderate (decrease by 1)	Moderate	Dependability: 9 of the 17 included articles had a dependability score of high. Credibility: significant equivocal.
The realisation of recovery in practice	Qualitative	High (no change)	Moderate (decrease by 1)	Moderate	Dependability: 9 of the 17 included articles had a dependability score of high. Credibility: significant equivocal.
Communication, relationships and decision‐making	Qualitative	High (no change)	Moderate (decrease by 1)	Moderate	Dependability: 9 of the 17 included articles had a dependability score of high. Credibility significant equivocal.
Family perspective of collaboration between themselves, the person with living experience, and the nurses					
Parents and partners/spouses identified helpful responses eg: respect and empathy but identified confidentiality as an issue	Qualitative				Unable to use ConQual approach as only one study was included
Adult children were least likely to experience positive responses from the mental health care system eg: memories from childhood	Qualitative				
Nurse perspective of collaboration between themselves, people with living experience, and their families					
The role of therapeutic engagement	Qualitative	Moderate (downgrade by 1 level)	Moderate (Downgrade by 1 level)	Low	Dependability: only 6 of the 13 included articles had a dependability score of high. Credibility: significant number of equivocal illustrations.
Impact of organisational factors	Qualitative	Moderate (downgrade by 1 level)	Moderate (downgrade by 1 level)	Low	Dependability: only 6 of the 13 included articles had a dependability score of high. Credibility: significant number of equivocal illustrations.

### 2.5 Data Extraction and Synthesis

A template was created by the researchers that reported key relevant characteristics of the studies including location, aim, methods, and participant characteristics. The corresponding author completed the initial data extraction, which was overseen and reviewed by the remaining authors.

Each included study was read several times to ensure that the research team understood the context and findings of the study. Key findings from each study were identified and tabulated including direct quotes attributed to the relevant member of the “Triangle of Care”. The practice domains in the National Framework for Recovery Oriented Mental Health Services (Australian Health Ministers' Advisory Council 2013, 6) (Table 1) were then used to group the articles according to the relevant domain; however, some articles addressed more than one domain.

**TABLE 1:** Practice domains for recovery‐focused care Australian Health Ministers' Advisory Council (2013) 6.

Domain 1: Promoting a culture and language of hope and optimism (overarching domain)			
Domain 2: Person first and holistic	Domain 3: Supporting personal recovery	Domain 4: Organisational commitment and workforce development	Domain 5: Action on social inclusion and the social determinants of health, mental health and wellbeing

The final stage of synthesis was to produce overall findings for each of the three groups, which were accompanied by an overall ConQual Score.

## 3 Results

### 3.1 Literature Search Results

**FIGURE 1:**
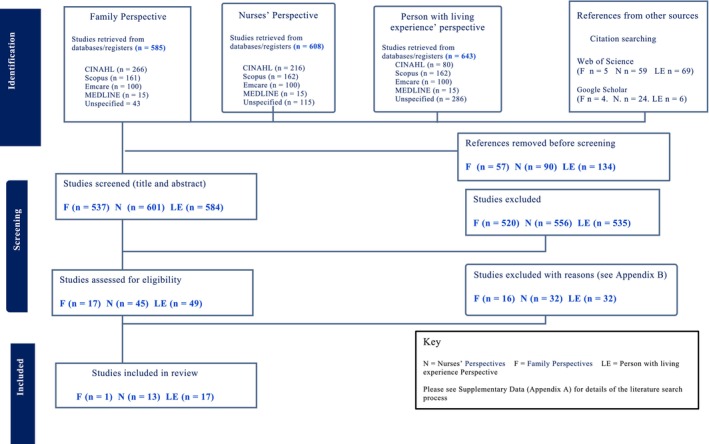
PRISMA diagram.

The literature search identified 718 potential papers from the lived experience group, 594 from the family group and 691 for the nurses' group (Figure 1). Once duplicates and title and abstract screening were completed, full text screening was conducted on 49 articles for the lived experience group, 17 for the family group and 45 for the nurses' group. After full text screening, a total of 17 for the lived experience group, one for the family group and 13 for the nurses' group were included in the systematic review. Figure 1 presents the PRISMA flow chart that summarises each step of the screening and selection process (Tricco 2018) for each group.

### 3.2 Study Characteristics

The 31 included studies were published between 2013 and 2023. Studies provided an international context, with studies related to the lived experience and nurses' perspectives predominantly from Australia (Wyder, Bland, et al. 2018; Wyder et al. 2016; Wyder, Bland, Herriot, and Crompton 2015; Wyder, Bland, Blythe, et al. 2015; Wilson et al. 2023; Tucker et al. 2020; Olasoji et al. 2020, 2018; McKenna et al. 2014; Lim et al. 2019; Isobel et al. 2021; Hristodoulidis et al. 2022; Gwinner and Ward 2015; Foster and Isobel 2018; Digby et al. 2020; Cleary et al. 2013; Bradley et al. 2021) and Europe (Waldemar et al. 2019, 2018; Van de Velde et al. 2021; Schön 2013; Moreno‐Poyato et al. 2021; Cheetham et al. 2018; Chambers et al. 2015; Ådnanes et al. 2018). The study providing the family perspective was conducted in the United States of America (USA) (Schaffer 2021).

The majority of studies explored the experience of an acute inpatient admission more generally (Ådnanes et al. 2018; McKenna et al. 2014; Jorgensen et al. 2020; Hristodoulidis et al. 2022; Haji Kassim et al. 2021; Cleary et al. 2013; Schaffer 2021; Wyder, Bland, et al. 2018; Wilson et al. 2023; Waldemar et al. 2019, 2018; Isobel et al. 2021; Eldal et al. 2019), while others focused on an involuntary admission (Wyder, Bland, Blythe, et al. 2015; Wyder et al. 2016), processes such as clinical handover (Olasoji et al. 2018, 2020; Van de Velde et al. 2021), and the management of particular behaviours [e.g., aggression and responses to distress and agitation by (Chambers et al. 2015; Tucker et al. 2020; Lim et al. 2019)]. While all studies used qualitative methodologies, a variety of methods were utilised, predominantly interviews and focus groups. See Table 2 for detailed characteristics of the included studies.

**TABLE 2:** Characteristics of included studies.

Author, date, country	Aim	Sample	Methodology	Methods
Ådnanes et al. (2018), Romania, Slovenia, Finland, Austria, Norway and Italy	To understand the multifaceted nature of psychiatric re‐hospitalisation	*n* = 55 in receipt of services for at least 1 year and had more than 1 hospitalisation	No methodology identified	Focus groups
Bradley et al. (2021), Australia	To explore the experiences of Aboriginal Australian women as they navigated the acute mental health inpatient journey	*n* = 11 with sufficient fluency in Standard English or Aboriginal English	Critical feminist, intersectional and de‐colonising methodologies	Interviews
Eldal et al. (2019), Norway	To explore how mental health patients experience current inpatient treatment in Norway	*n* = 14 (11 of the 14 were interviewed twice) voluntary treatment for at least 3 weeks, no active psychosis	Hermeneutic phenomenology	Interview
Huang et al. (2020), China	To explore the perceptions of shared decision‐making from the perspective of people diagnosed with schizophrenia in China	*n* = 12 diagnosis of schizophrenia for more thana 1 year	Qualitative descriptive	Interviews
Isobel et al. (2021), Australia	To explore the perspectives of consumers of mental health services in Australia and their family members in relation to providing a trauma‐informed service	*n* = 10 who were current or previous recipients of care from mental health services	Experience‐based co‐design, drawing on participatory action research	Focus groups
Moreno‐Poyato et al. (2021), Spain	To explore the perspective of people with experiences in acute mental health units to the intervention “Reserved Therapeutic Space”	*n* = 11 who were hospitalised within the previous 2 years, excluded people who had been admitted within the previous month	Qualitative descriptive design	Focus groups
Olasoji et al. (2018), Australia	To explore the views of consumers with a mental illness, who have not had prior involvement in nursing handover, about their need to be involved in nursing handover	*n* = 11 who had been admitted for a minimum of 5 days prior to interview	Exploratory descriptive qualitative design	Interviews
Olasoji et al. (2020), Australia	To explore the views of consumers with a mental illness about their experiences of being involved in nursing handover following the implementation of a change in nursing handover that involved consumers.	*n* = 10 who had been admitted for a minimum of 5 days prior to interview, returned to unit 6 months after implementation of living experience involvement in handover	Qualitative exploratory descriptive design	Interviews
Ould Brahim et al. (2020), Canada	To determine which actual and/or potential nursing interventions, attitudes, actions, or behaviours are perceived as helpful by psychiatric inpatients with a dual diagnosis	*n* = 12 from 3 different inpatient units—psychosocial rehabilitation, acute psychosis and mood disorders	Qualitative descriptive design	Interviews
Schön (2013), Sweden	To explore gender differences of the impact of compulsory inpatient care on recovery from severe mental illness	*n* = 30 (15 men and 15 women) diagnosed with a severe mental illness, not hospitalised within the past 2 years	Grounded theory used to analyse narratives	Interviews
Van de Velde et al. (2021), Belgium	To map the experiences of consumers regarding nursing handover involving consumers	*n* = 13 during a voluntary admission and experienced handover at least 3 times	Phenomenology	Interviews
Waldemar et al. (2018), Denmark	To explore whether these recovery‐orientation efforts are in any way reflected in the inpatient experience of care and treatment	*n* = 14 with a range of diagnoses and excluded forensic admission	Descriptive study design	Interviews
Waldemar et al. (2019), Denmark	To explore how recovery‐oriented practice is reflected in the interactions between patients and health professionals regarding treatment	21 days over 84 h in total on both a locked intensive ward and open ward	Ethnography	Participant observation and informal interviews
Wilson et al. (2023), Australia	To investigate user perspectives to inform the redesign of a rural and regional facility	3 groups with a range of professionals including doctors, nurses and allied health (total 27). 1 group of 4 consumers 3 groups of carers (total 5)	No methodology identified	Focus groups
Wyder, Bland, Herriot, and Crompton (2015), Australia	To describe the experience of interactions with health care professionals when admitted under an involuntary treatment order	*n* = 25 people on an endorsed treatment order who were close to discharge	No methodology identified	Interviews
Wyder et al. (2016), Australia	To explore the tensions between the principles of recovery‐oriented care and control and involuntary treatment	*n* = 25 who were admitted under an endorsed treatment order in a large metropolitan context	No methodology identified	Interviews
Wyder, Roennfeldt, et al. (2018), Australia	To present a collective and collaborative reflection of experience of mental health challenges and recovery	*n* = over 20 individuals	Within the traditions of creative thesis and analytic autoethnography	Creative artefact and academic commentary/critical reflection
*Family member perspective*				
Schaffer (2021), United States of America	To explore how parents, partners or spouses, siblings, and adult children experienced interactions with mental healthcare providers and staff	5 parents 5 partner/spouse 5 siblings 5 adult children Their relative had a bipolar disorder diagnosis	Descriptive qualitative research	Interviews
*Nurses' perspectives*				
Chambers et al. (2015), United Kingdom (England)	To explore the thoughts and feelings experienced by English MHNs when caring for and managing distressed and/or disturbed service users	*n* = 12 from 3 different acute units	No methodology noted	Focus groups
Cheetham et al. (2018), United Kingdom (England)	To contribute towards understanding how discourses influence relating on psychiatric wards	6 staff members participated in focus groups and 3 staff members interviewed—only 3 of these staff were nurses	Foucauldian discourse Analysis	Interviews and focus groups
Cleary et al. (2013), Australia	The explore the understanding of acute mental health nurses of recovery, how it is incorporated in practice and identify practical realities that facilitate or hinder its implementation	*n* = 21from 4 different acute units	No methodology noted	Interviews
Digby et al. (2020), Australia	To evaluate psychiatric staff experiences and attitudes relating to Psychiatric Behaviours of concern (Psy‐BOC)	5 Focus groups—2 groups clearly identified as having nurses only—12 nurses in total	Qualitative descriptive design	Focus groups
Foster and Isobel (2018), Australia	To explore nurses' practices and perspectives on working with families while using family rooms	20 nurses—3 acute units and 1 rehabilitation setting	Exploratory descriptive	Interviews
Gwinner and Ward (2015), Australia	To understand how a PICU (Psychiatric Intensive Care Unit) applies a recovery approach	*n* = 45 from 2 large metropolitan hospitals	No methodology identified	Focus groups and literature review afterwards
Haji Kassim et al. (2021), Brunei Darussalam (Malay)	To explore the perceptions of psychiatric nurses' regarding the reasons for readmission and their role in reducing readmission	5 focus groups—total of 24 nurses (must have had more than 1 year work experience in “psychiatric department”)	Qualitative descriptive design	Semi‐structured interview using Focus Group Discussion
Hristodoulidis et al. (2022), Australia	To examine the experience of nursing practice, understanding and knowledge of personal recovery	Total of 7 = 6 registered nurses and 1 enrolled nurse	Hermeneutic phenomenology	Interviews
Jorgensen et al. (2020), Denmark	To explore how recovery is constructed and which conditions for the possibilities to promote recovery are expressed by nurses	10 nurses (5 inpatient and 5 outpatient)	Social constructivism and critical discourse analysis	Interviews and patient records
Lim et al. (2019), Australia	To understand nurses' knowledge of the components of recovery, and how it can be utilised to reduce aggression	*n* = 27 members of the Australian College of Mental Health Nurses across Australia	Exploratory study using constructivist grounded theory” p. 238	Interviews
McKenna et al. (2014), Australia	To describe current practice and determine the extent to which elements of existing nursing practice resemble the domains of recovery‐oriented care	5 focus groups—total of 46 nurses Mental health services not yet required to use the recovery‐oriented model	Exploratory research design	Focus group interviews
Tucker et al. (2020), Australia	To understand mental health nurses' experiences in recognising and managing agitation among inpatients and relate them to best practice principles	4 focus groups with 5 nurses in each (total *n* = 20)	Cross‐sectional descriptive	Focus groups
Waldemar et al. (2019), Denmark	To explore how recovery‐oriented practice is reflected in the interactions between patients and health professionals regarding treatment	Some participants were court ordered however ward was not part of forensic system Health professionals were from a variety of roles 84 h total observations over 21 days	Ethnography	Participant observation and informal interviews

### 3.3 Quality Assessment

All of the studies received a “yes” score for Question 10, that the conclusions flowed from the analysis and interpretation of data. In only one of the 31 studies (Chambers et al. 2015) it was unclear if the methodology and methods were congruent because the methodology was not identified by the authors. Data collection methods were primarily focus groups or interviews. Nine of the 17 articles from the lived experience group (Wyder, Bland, et al. 2018, Wyder et al. 2016; Wyder, Bland, Herriot, and Crompton 2015; Van de Velde et al. 2021; Ould Brahim et al. 2020; Moreno‐Poyato et al. 2021; Huang et al. 2020; Eldal et al. 2019; Bradley et al. 2021), the one article from the family group (Schaffer 2021) and six from the nurse group (Waldemar et al. 2019; Tucker et al. 2020; Lim et al. 2019; Hristodoulidis et al. 2022; Cleary et al. 2013; Cheetham et al. 2018) had a high dependability score. Refer to Appendix S3 for details. Two from the nurse group have low dependability (Gwinner and Ward 2015; Chambers et al. 2015). The main concerns regarding dependability were a lack of reporting on the theoretical and cultural position of the researcher, and the bi‐directional influence of the researcher on the research. These aspects potentially limit the dependability of the findings by obscuring the influence of the researcher on the research, and therefore power imbalances during the conduct of the studies. The majority of findings in the synthesis were either unequivocal or equivocal, adding to the overall strength of the findings.

### 3.4 Review Findings

To promote conceptual clarity in the remainder of this paper, the term “recovery” will be used to refer to personal recovery unless specifically stated otherwise. After aligning the review results with the five practice domains (Australian Health Ministers' Advisory Council 2013, 6), most of the included articles focused on the provision of person‐first and holistic care, supports for personal recovery, and aspects of organisational commitment and workforce development. There were a limited number of papers related to the overarching domain of promoting hope, using recovery‐focused language and addressing the socioeconomic factors that impact on mental health recovery. This is important to note because for people with mental health challenges, having nurses that support connection with their spirituality, or their community and cultural practices, can be an important aspect of promoting their personal recovery journey and helping to provide a sense of hope, connection, and identity (Ramluggun et al. 2021; Baxter et al. 2022).

Table 3 below presents the key themes representing the perspective of each group separately. The five domains of Recovery‐Focused Care (Australian Health Ministers' Advisory Council 2013, 6) (Table 1) were used as the framework.

**TABLE 3:** Overview of themes.

People with living experience	The experience of a mental health admission The realisation of recovery in practice Communication, relationships and decision‐making
Family	Parents and partners/spouses identified helpful responses e.g., respect and empathy but identified confidentiality as an issue Adult children were least likely to experience positive responses from the mental health care system eg: memories from childhood
Nurses	The role of therapeutic engagement Impact of organisational factors

#### 3.4.1 Perspectives of People With Lived Experience of Mental Health Challenges

A total of 55 themes including 63 illustrations were used for this synthesis. From the lived experience group 10 of the 17 included articles referred to aspects of person first and holistic care (Domain 2) (Waldemar et al. 2018; Schön 2013; Ould Brahim et al. 2020; Olasoji et al. 2020, 2018; Isobel et al. 2021; Huang et al. 2020; Eldal et al. 2019; Bradley et al. 2021; Ådnanes et al. 2018), 8 referred to supporting personal recovery (Domain 3) (Wyder, Bland, et al. 2018, Wyder et al. 2016; Wyder, Bland, Herriot, and Crompton 2015; Wilson et al. 2023; Waldemar et al. 2018; Van de Velde et al. 2021; Moreno‐Poyato et al. 2021; Bradley et al. 2021) and 12 referred to organisational commitment and workforce development (Domain 4) (Isobel et al. 2021; Wyder, Bland, et al. 2018, Wyder et al. 2016; Wyder, Bland, Herriot, and Crompton 2015; Wilson et al. 2023; Waldemar et al. 2019, 2018; Van de Velde et al. 2021; Ould Brahim et al. 2020; Olasoji et al. 2020, 2018; Moreno‐Poyato et al. 2021). Refer to Table 1 above and Appendix S5 for details. From analysis of these groupings, three key themes emerged: (1) the experience of a mental health admission, (2) the realisation of recovery in practice, and (3) communication, relationships and decision‐making.

##### 3.4.1.1 The Experience of a Mental Health Admission

People with lived experience of mental health challenges sometimes find admission to hospital an opportunity to make sense of recent events. Participants described three factors as either beneficial or a hindrance to recovery whilst in hospital: having a place of safety, feeling connected to others, and having a sense of autonomy and control (Wyder, Roennfeldt, et al. 2018). Participants in the study by Ådnanes et al. (2018) reported that admission may provide a sense of relief and acceptance with at least an invitation to participate in decision‐making (Waldemar et al. 2019), with some reporting that subsequent admissions were less traumatic than their initial one. One participant stated

Yes, it was a relief that I went, like I was going to a vacation. So, I can rest my brain and pull myself together and then forward again (Ådnanes et al. 2018, 519)

Some were disappointed in their treatment and that it lacked purpose, while others saw it as inevitable (Ådnanes et al. 2018). In the only study providing a critical cultural focus on personal recovery, the Indigenous Australian women in the study by Bradley et al. (2021) reported a sense of loss of control and isolation from family and community, leaving them reliant on others to “tell their story”. Whilst participants wanted information about their treatment, they also described nurses as avoiding contact with them and “uncaring” about their personal and cultural concerns (Bradley et al. 2021, 922).

Isobel et al. (2021) highlighted from the lived experience perspective the role of active engagement and communication when providing trauma‐informed care, for example discussing with a nurse what they would like included in their clinical notes, and active efforts to provide a sense of safety through engagement. People with comorbid mental health challenges and substance use disorder highlighted the importance of therapeutic relationships with nurses to implement a variety of helpful nursing practices including promotion of their physical health, providing support with psychosocial well‐being, identifying the relationship between drug and alcohol use and mental health (Ould Brahim et al. 2020). While admission to hospital needs to provide safety and security for someone who feels vulnerable, experiences of shame and enforced socialisation can negatively impact recovery (Eldal et al. 2019). Wilson et al. (2023) explored the effects of the physical environment on people admitted to mental health units which promoted physical security (such as room design), relational security (private spaces to talk, needing to ask permission to access certain areas of the ward) and procedural security (promoting efficient workflow, promoting diversity, managing acuity, and including family and people with lived experience in mental health care).

While involuntary admissions to mental health units and involuntary treatment orders (ITO) can be disempowering for some, the experience also has the potential to benefit the recovery of people with lived experience. Staff communication skills, behaviours and attitudes were highlighted as key for shaping the experiences of the person and their family during this time (Wyder, Bland, Blythe, et al. 2015). People with lived experience on an ITO still wanted to feel connected to staff, receive information and provide input, and work in partnership (Wyder, Bland, Herriot, and Crompton 2015). Critical to this is the promotion of a sense of safety, individual agency and empowerment (Wyder et al. 2016). Many participants in the study by (Schön 2013) described a lack of treatment within the inpatient unit. The majority of participants did not know which treatment to expect; however, women generally described treatment absence as more of an emotional loss than men did. While medication was described by participants of both genders as essential to recovery, it was reported as one of the most commonly forced interventions in involuntary care contexts (Schön 2013). Several women described a sense of helplessness regarding how medication was administered, describing this process as “traumatic” and eroding trust in “ward staff” of unreported designation. Enforced medication was as common for men. Men generally appeared more resigned to following the “institutional order” albeit still disappointed by a lack of treatment (Schön 2013).

##### 3.4.1.2 The Realisation of Recovery in Practice

Two articles identified how recovery principles were implemented and experienced in practice. Waldemar et al. (2018) reported that admission can provide a feeling of acceptance and facilitate social contact; however, it can feel predominantly medication‐focused and longing for more contact with nursing staff. Admission can leave people feeling like they are uninformed, with limited choices while under constant observation and assessment (Waldemar et al. 2018). Interactions between people with lived experience and health professionals were described “as if”, meaning they appeared “shallow or artificial” (Waldemar et al. 2019). In this style of interaction, limited choices are provided and often competing demands, for example, bed pressure and lack of continuity of staff impact collaboration and decision‐making. Waldemar et al. (2019) describe how

A male patient goes to the closed office door and knocks. Nurse Jane opens the door and the patient says to her that he'll agree to take Antabuse, and wants to take the next bus home. The nurse tells him that she'll let the doctor know. The patient then says that he doesn't really want to take it, but that he'll do it in order to go home, because he feels uncomfortable here. (Waldemar et al. 2019, 324)

##### 3.4.1.3 Collaboration, Building Therapeutic Relationships and Shared Decision‐Making

Various aspects of communication and the importance of therapeutic relationships have been highlighted by people with lived experience of mental health challenges. Participation in treatment, and the provision of information about that treatment, are key aspects of developing recovery‐oriented practice. While participants in one study (Huang et al. 2020) wanted their families to be involved with shared decision‐making, they preferred medical staff to make final treatment decisions. Specifically, participants did not want mental health nurses involved, because nursing was in the “caring profession” and were “subordinate” to physicians (Huang et al. 2020, 850). Participants in the study by Moreno‐Poyato et al. (2021) found the intervention “Reserved Therapeutic Space”, that is the specific creation of a time and space with nurses that is dedicated to the expectations and needs of the person, to be helpful. However, the availability of nurses, and the manner in which the intervention was delivered (rather than the content), was important to participants as was the effectiveness of the intervention.

Three studies described the involvement of people with lived experience in nursing handover or hand‐offs (Van de Velde et al. 2021; Olasoji et al. 2020, 2018). Connection with a nurse, particularly in the first moments of admission, was found to be a valuable opportunity for introductions to be made, an explanation of how people with lived experience can be involved in this communication process, and commence this phase of their recovery (Van de Velde et al. 2021). Involvement of people with lived experience in these processes provided opportunities for them to participate in their care by knowing who their allocated nurse was if they had any questions or concerns and provided an opportunity to ask questions or clarify information about their experience and care.

The difference was that in the beginning I was not sure exactly they were doing. What should I expect? But they did take their time to explain that briefly. And after a week or a week and a half I started to understand it very well and I started to feel the need for that moment. (Van de Velde et al. 2021, 1719)

This can be empowering for people with lived experience as it actively includes them, promotes shared decision‐making, and provides accessibility to nurses. Involvement in these processes may also provide an opportunity for family members to participate and advocate for their family member (Olasoji et al. 2020, 2018).

#### 3.4.2 Perspectives of Family Members

Only one study that explored the family experience of engagement with nursing staff and their loved one with mental health challenges met the inclusion criteria for this review (Schaffer 2021). Four illustrations of data used for discussion are included in Appendix S4.

The unique aspect of the study by Schaffer (2021) was the exploration of the experiences of people with different family roles (sibling, parent, adult child, and partner/spouse). While this study explored mental health services in general, within the inpatient context family appreciated an empathic response and raised issues related to managing unexpected discharges from the hospital, while adult children shared and reflected on their memories from childhood with an unwell parent and a lack of supportive response from hospital staff:

A woman who was trying to convince staff that her partner was not ready for discharge from a mental health unit, resorted to recording a middle‐of‐the‐night phone call from him to provide evidence of concerning symptoms to the psychiatrist. (Schaffer 2021, 1551)

Concerns related to medication were raised by the partner/spouse groups and the adult children including finding the right medication, side effects of medication or overmedication. While all groups experienced challenges navigating the mental health system, parents and spouses/partners tended to encounter more challenges such as confidentiality and receiving education. Mental health nurses can help promote positive affirmation of the family role and their contribution and are encouraged to promote attention to both informational and emotional and social support of family members.

#### 3.4.3 Perspectives of Nurses

Seven of the 13 included studies that discuss the nurses' perspectives were situated within an Australian context (Tucker et al. 2020; McKenna et al. 2014; Lim et al. 2019; Hristodoulidis et al. 2022; Gwinner and Ward 2015; Foster and Isobel 2018; Digby et al. 2020). The remaining six were from the United Kingdom, Denmark, Norway, and Brunei Darussalam (Waldemar et al. 2019; Jorgensen et al. 2020; Haji Kassim et al. 2021; Cleary et al. 2013; Cheetham et al. 2018; Chambers et al. 2015). All 13 articles presented findings of qualitative studies. Six of the 13 articles received a dependability score of high (Cheetham et al. 2018; Cleary et al. 2013; Hristodoulidis et al. 2022; Lim et al. 2019; Tucker et al. 2020; Waldemar et al. 2019), five moderate (Digby et al. 2020; Foster and Isobel 2018; Haji Kassim et al. 2021; Jorgensen et al. 2020; McKenna et al. 2014), and one, low (Gwinner and Ward 2015). There were 43 themes in total within the included studies, with 46 illustrations used to conduct this synthesis. Six of the 13 articles related to person‐first and holistic care (Domain 2) (Chambers et al. 2015; McKenna et al. 2014; Haji Kassim et al. 2021; Gwinner and Ward 2015; Foster and Isobel 2018; Cleary et al. 2013), 11 related to Supporting personal recovery (Domain 3) (Chambers et al. 2015; Cheetham et al. 2018; Cleary et al. 2013; Digby et al. 2020; Foster and Isobel 2018; Gwinner and Ward 2015; Haji Kassim et al. 2021; Hristodoulidis et al. 2022; Lim et al. 2019; McKenna et al. 2014; Tucker et al. 2020), 10 to organisational commitment and workforce development (Domain 4) (Chambers et al. 2015; Cleary et al. 2013; Digby et al. 2020; Foster and Isobel 2018; Gwinner and Ward 2015; Hristodoulidis et al. 2022; Jorgensen et al. 2020; Lim et al. 2019; Tucker et al. 2020; Waldemar et al. 2019), and two related to social inclusion and social determinants of mental health and wellbeing (Domain 5) (Cleary et al. 2013; Jorgensen et al. 2020). Two key themes were identified from the perspective of nurses: the role of therapeutic engagement and the impact of organisational factors.

##### 3.4.3.1 The Role of Therapeutic Engagement

Ten papers described various aspects of the role of the therapeutic relationship between nurses and people with lived experience of mental health challenges (Chambers et al. 2015; Cheetham et al. 2018; Cleary et al. 2013; Digby et al. 2020; Foster and Isobel 2018; Gwinner and Ward 2015; Lim et al. 2019; McKenna et al. 2014; Tucker et al. 2020; Waldemar et al. 2019). Admission to an acute mental health inpatient unit, often involuntarily and into an environment that may be perceived as unpredictable, can be a critical time in the recovery process. Therefore, how nurses approach recovery‐oriented practice and provide support during this time can be influential for recovery. Supporting people who are “distressed and/or disturbed” within the inpatient setting has been found to provoke negative feelings in nurses such as fear, anxiety, and vulnerability (Chambers et al. 2015). Nurses reported experiencing cognitive dissonance, for example, in the application of policies and procedures, and conflict between benevolence and malevolence if coercion, such as enforced behaviour or treatment, was required, fearing damage to the therapeutic relationship. Some nurses noted that an advanced care directive may be helpful, but having the time to do so was a limitation. Support from colleagues and the broader organisation, for example, through debriefing, was noted as important.

Aggression and agitation are often seen on acute inpatient units and are often a sign of distress that can lead to more restrictive measures being utilised (Digby et al. 2020). Tucker et al. (2020) and Digby et al. (2020) have described the recognition and management of agitation in people admitted to acute mental health units. Tucker et al. (2020) reported that recognition of agitation involves self‐awareness and self‐reporting of the symptoms of agitation in those with living experience as well as the importance of the knowledge and clinical assessment skills of nurses and effective communication within the team. The management of agitation should be flexible and individualised, requires rapport, and can be pharmacological or not. Management of more significant agitation may require the involvement of a Psychiatric Behaviours of Concern (Psy‐BOC) Response Team (Digby et al. 2020). The Psy‐BOC team is similar to a medical rapid response team called to a ward to assist in response to medical deterioration. Identifying deterioration and individual early warning signs of Psy‐BOC are key factors in the management of behaviours of concern. How well known the person is, or is not, impacts the capacity of the team to identify triggers and implement appropriate management of agitation. More experienced nurses may be more skilled at using therapeutic engagement and effective communication to help bring a timely resolution.

Features of the ward environment are also influential in enhancing therapeutic engagement such as the provision of comfort and privacy which may help de‐escalate agitation or distress (Digby et al. 2020). Some nurses also expressed reservations or concerns about using a Psy‐BOC team, and whether the intervention may increase the risk of harm for staff and negatively impact communication within the treating team when the Psy‐BOC team leaves. Lim et al. (2019) discussed how recovery‐oriented principles through early intervention, the identification of triggers and the reason for the behaviour, can reduce the use of more coercive interventions. Nurses who reframed aggression as a learning opportunity and provided feedback to people with lived experience were identified as reducing the risk of future aggression (Lim et al. 2019). This approach creates an opportunity for therapeutic engagement to facilitate learning and skills development which is an important aspect of recovery.

Waldemar et al. (2019) focused on the distinction between a theoretical understanding of recovery and how this is acted out in practice by using participant observation, with the authors reporting interactions rather than providing a direct first‐person voice of nursing staff. While the two wards included in this study were both general acute mental health, some people had forensic backgrounds. In practice, collaboration with patients was described “as if”, indicating that communication had a shallow quality that “subjugated” the needs of patients to other competing demands. Discourses that are “medical‐technical‐legal” and “ordinary humane relating” hold competing implications for nurses who are relating with/engaging with people with lived experience of mental health challenges. The former and more dominant discourse implies status and power, technique and objectivity while the latter features care and compassion for another that is not contingent on technique. While tensions are found between these two, a third called “collaborative exploration” privileges purposeful ways of relating that are therapeutic and provides a more equal power relationship and offers containment and hope (Cheetham et al. 2018). The conditions for someone's personal recovery can be demonstrated in practice in a manner more akin to clinical recovery; however, there remains an expectation of self‐responsibility (Jorgensen et al. 2020).

Three studies highlighted the importance of focusing on strengths when engaging with people with mental health challenges. Focussing on strengths and already developed resources and supports can support people to de‐escalate their own behaviour, empower them, and instil trust and hope (Lim et al. 2019). Nurses in the study by McKenna et al. (2014) identified a focus on strengths as a way of developing rapport.

I try to identify one strength that I see in them and say, “Oh, that's fantastic!” In the conversation I find they have a hobby or something, and I focus on that, and say how great it is that they do such and such. It also builds rapport (McKenna et al. 2014, 530)

They highlighted successful outcomes of previous admissions as strengths and to develop hope for the future. Nurses supported people to develop strategies to achieve goals.

Gwinner and Ward (2015) conducted focus groups, which they augmented by conducting a literature review. Their results highlight how the storytelling, or therapeutic relationship that can develop through engagement, can be used to encourage people to draw on their current resources. The study by McKenna et al. (2014) highlighted the importance nurses placed on promoting hope, utilising individual strengths and taking opportune moments to engage and develop rapport as there was not always time for more meaningful engagement.

Three of the 13 papers discuss the nurses' perspective of working with families. Support, particularly from family, was identified by some nurses in the study by Cleary et al. (2013) as an important aspect of recovery. McKenna et al. (2014) highlight how nurses proactively include family members, for example in meetings or peer support, can contribute to holistic care. Foster and Isobel (2018) described the nurse's perspective of working with parents of minors, their families and the use of family rooms on the ward. Nurses recognised the importance of maintaining family relationships for recovery, however their main concern was their need to balance management of risk and safety, for example assessing the mental state of the adult/parent and interactions with family members and others on the ward. Nurses were less focused on the therapeutic opportunities provided by family visits; however, they did recognise the practical support they can provide as well as the cultural expectations of the family. While the study by Foster and Isobel (2018) provided valuable insight into the family influence on recovery, it must be noted that the perspectives of nurses working on an acute ward could not clearly be separated from those working on a rehabilitation ward based on data provided in the paper; however, in some instances, the context could be inferred, for example, from the context of quotes within the narrative.

##### 3.4.3.2 Impact of Organisational Factors

The other key theme was the influence of organisational structure and the ward milieu on the use of recovery‐oriented principles in clinical practice. Nurses commonly referred to the prevailing biomedical model, which was demonstrated in the use of medication to manage agitation or aggression and the language used in either clinical notes or conversations (Cleary et al. 2013; Hristodoulidis et al. 2022). The most prominent term that nurses used to describe recovery was “holism” (Cleary et al. 2013). They did, however, describe a medical understanding of recovery and practical realities such as a short length of stay that may present recovery as more rhetoric than practical reality (Cleary et al. 2013).

The study by Gwinner and Ward (2015) within the Psychiatric Intensive Care Unit (PICU) environment highlighted the “safeguarding” aspect of the nurse's role, to ensure safety of everyone. This aligns with nurses in the study by Hristodoulidis et al. (2022) referring to taking on a custodial role and the study by Foster and Isobel (2018) emphasising risk aversion and safety management. In relation to the model of nursing care provided, McKenna et al. (2014) refer to the importance of a primary care model, where one nurse gains particular knowledge of the needs of a person, and the importance of continuity of care between the inpatient and the community teams, such as the case manager visiting before discharge. Continuity of care is also referred to within the inpatient environment itself to help facilitate rapport development (Hristodoulidis et al. 2022), as was the need for more education, for example in the management of agitation (Cleary et al. 2013).

Nurses also made reference to the influence of policies and procedures and the provision of education, debriefing, and supervision. For example, nurses in the study by (Digby et al. 2020) referred to the role of the Psychiatric Behaviours of Concern Team. One criticism was the relatively short duration of the intervention, the lack of communication, and missed educational opportunities for staff. Nurses also referred to this team leaving nurses feeling devalued in their role and their experience and prior knowledge of the person being relatively unimportant.

## 4 Discussion

This systematic review simultaneously explored the perspectives of people with lived experience of mental health challenges, their family members, and mental health nurses regarding collaboration about mental state changes during an inpatient admission.

A key concept identified by all three members of the “Triangle of Care” was the importance of ongoing communication between the members. Studies such as those conducted by Isobel et al. (2021) and Waldemar et al. (2018) highlighted that people with mental health challenges want to be heard respectfully and to receive holistic care from nurses that is inclusive of their identified family. This aligns with previous findings of Waller et al. (2019) and Wonders et al. (2019). People with lived experience Waller et al. (2019) expressed the need for staff to instigate discussions with family about their needs while stressing the importance of boundaries regarding family involvement Waller et al. (2019). Similarly Wonders et al. (2019) concluded that choice regarding family involvement is critical: family involvement needs to be accessible, family need to be willing to engage, and people need choice about who is involved, how they are involved and when they are involved. Wyder, Roennfeldt, et al. (2018) focus on the importance to families of being kept informed. Doody et al. (2017) also highlight the complexities of communication from the family perspective including confidentiality that can leave families feeling excluded and subject to less coordinated care. Nurse communication skills have been identified as critical in demonstrating empathy, managing challenging interactions and providing safety (Cleary et al. 2012; Kanerva et al. 2015). However literature even recently demonstrates strategies used to facilitate (such as utilising strengths) and the challenges for example with maintaining boundaries (Xiang et al. 2025).

Indicative of a more personal recovery‐focused approach, this review highlighted that people admitted to mental health units want to feel empowered and to be given actual choice in their treatment and care planning, although this does vary in practice partly due to acuity (Waldemar et al. 2018; Wyder, Roennfeldt, et al. 2018). Whether the admission is voluntary, involuntary, the first, or subsequent admission are all other factors influencing the level of collaboration (Ådnanes et al. 2018). Consistent with the broader literature, family members want their role and experience acknowledged and welcomed; however, confidentiality was raised as one impediment to this (Muddle et al. 2024). Nurses often wanted to work in a recovery‐oriented way, with the wider literature highlighting some of the factors impacting on their work, for example, the complexity of their role, time, workload, and operational demands (Wyder et al. 2017).

Family members however, continue to have experiences like those described earlier by Schaffer (2021). Family members understood and wanted to support a personal recovery approach that prioritises relationships (Poon et al. 2025). However, the literature demonstrates how families feel disillusioned with mental health services, that their needs are not recognised, and feel they are the “invisible experts” (Abou Seif et al. 2022). This presents mental health services, ward leadership, and each nurse with the obligation to improve the focus and outcome of every interaction. These are opportunities to share information and education and provide emotional and practical support. This leaves families and people with mental health challenges feeling more supported and more capable of identifying and utilising strengths, supports and resources for both family and personal recovery.

Existing literature demonstrates that the physical environment of an acute mental health unit, for example locked doors, can be countertherapeutic for people admitted and detrimental for staff, for example reduction in freedom for people admitted to the unit and conflict between a recovery and more restrictive role (Searby et al. 2023). Issues related to power differentials and control in favour of nurses and medical staff have long been reported and discussed (Cleary 2003; Johansson et al. 2006). This literature review, reflective of more recent studies and a shift towards personal recovery and shared decision‐making, still describes how power dynamics reflective of a historical biomedical approach and separation from a familiar home and family context, however, can be experienced as disempowering of communication within the “Triangle of Care” (Wilson et al. 2023).

This literature review highlighted that some of these factors are related to the context of the inpatient setting, particularly within a high dependency context. These include the need for design of a safe ward while providing nurses with safe and private areas for discussions with the person with lived experience and an area for family to visit where they feel safe and comfortable and potentially engage with nursing staff. Involving and including people with lived experience, and potentially family members in nursing handover, is another potential to improve communication and engagement. For people with lived experience this provides some knowledge of who they could speak to if they need support; however, again privacy and practical issues were raised for consideration.

Attitudes, education and experience of nursing students and mental health nurses were identified as influential on the day‐to‐day interactions with people with lived experience and their families, the management of mental state deterioration, and the recovery process in the broader sense (Gyamfi et al. 2020; Happell et al. 2019). The experience of a first admission may be different from that of any subsequent admissions. Subsequent admissions may be perceived more positively if recovery is understood as a non‐linear process. If family members perceive stigma, this could potentially create a communication barrier between themselves, the person with lived experience, and nursing staff. The nurse's understanding of what mental health recovery means, and how that is operationalised in clinical practice day to day, is influenced by many factors. These factors include the perceived or actual dominance of the traditional medical model and the capacity of the person with mental health challenges to engage (especially when someone is in the acute phase of mental health challenges), time pressures, and resources. There are also cultural factors to consider, such as the importance of connection to, and involvement of, family (which may be extended depending on the cultural background) (Panadevo et al. 2025).

There is a potential power differential between the person with lived experience and nursing staff, particularly during periods of high acuity mental health challenges. This may arise from involuntary admissions that include the application of mental health legislation, seclusion and/or restraint, locked doors and restrictions on freedom. Family/friends may also experience heightened emotion related to the admission, and there may be an ongoing impact on family relationships. Nurses also work within a service/system that necessarily has its own policies and guidelines. This, however, may be perceived by nurses as limiting their autonomy and opportunity for therapeutic practice, leaving them more analogous to a custodial role. An approach more akin to a biomedical and more restrictive approach may be utilised, including seclusion and restraint, involuntary admission, and restrictions on leave from the unit.

Capacity to participate in decision‐making may be considered by staff as limited or non‐existent, particularly during periods necessitating acute admission. Providing nursing care during this period can raise challenging emotions for nursing staff, perhaps more so if a Psychiatric Behaviours of Concern Team was required to intervene. This does not, however, necessarily mean that people with lived experience are excluded from involvement in decision making about their care. Opportunities to do this include an “advance directive” outlining preferences for care prior to acute mental state deterioration and debriefing for both people with lived experience and staff after a seclusion event. Nurses can and do want to try alternative de‐escalation techniques, such as using rapport that has developed between them and the person in a distressed or agitated state or using PRN (as required) medication before using more restrictive measures.

This review has identified areas for further research. The lack of cultural diversity in the review may not be reflective of the views of other cultural groups, nor individuals representative of any particular family role. The study by Muddle et al. (2024) used family members rather than unpaid carers more broadly, which is another area for further exploration.

It was noteworthy that this literature review highlighted the value of identifying strengths and resources of people with mental health challenges (Lim et al. 2019; McKenna et al. 2014). Strengths can be identified and provide an opportunity to improve emotional regulation, which can be included in care plans or safety plans, with the aim of averting instances of heightened agitation and restraint and seclusion. Working with the family and individuals holistically may enable nurses to recognise and build on the strengths and resources of the family.

Nurses highlighted the need for improved organisational structures and supports to further facilitate recovery‐focused work that encourages communication between all members of the Triangle of Care. This included the provision of clinical supervision and debriefing after incidents of aggression, to both manage the emotional response of nurses and people who are subject to restrictive practices, as an opportunity for learning and to promote recovery.

This literature review presented the perspectives of the Triangle of Care about collaboration between them during a mental health admission. This should be individualised, recognising triggers and strengths and resources to help de‐escalate and aim to avert more restrictive practices. To do this, the ward environment needs to be conducive, for example allowing time for 1–1 discussions in a safe and therapeutic environment. People with mental health challenges should be provided with choice about who to include and how, while nurses are provided with a supportive structure including professional education and support.

While the findings of this literature review focused on the management of agitation, what is noteworthy is the limited discussion of deterioration in mental state related to self‐harm and suicidal ideation in relation to recovery‐focused nursing practice. Suicidal ideation and the decision to act on those thoughts can be considered to represent an existential challenge that is associated with the expression of ambivalence, disconnection, shame, and feelings of worthlessness and lack of purpose in life (Sellin et al. 2017). Self‐harm can be framed as emotional distress that is an expression of challenges coping with the difficulties of life (Morrissey et al. 2018). How nurses identify and respond to suicidal ideation and self‐harm, and how risk is assessed and managed, is critical. Recovery‐focused nursing care involves moving from a biomedical frame of “expert” or taking a critical response to a relational and collaborative response within the Triangle of Care to create a shared understanding of changes and explore the meaning behind the behaviour, to provide a space for personal expression, and to help promote a sense of belonging, purpose, and connection.

There was a lack of recognition within the literature of taking a recovery‐focused approach to the identification and management of anxiety and depression within the inpatient context. This has been recognised within the broader literature (Vicent‐Gil et al. 2023; Hurtado et al. 2020; Fernandez et al. 2023) and could benefit from further exploration. Researchers have urged clinicians to attend to both objective and subjective experiences and to consider and provide psychological support in addition to medication (Hurtado et al. 2020). In relation to depression, helping to maintain hope as one aspect of recovery and the attitudes of family and the workplace can be an important factor (Fernandez et al. 2023).

## 5 Strengths and Limitations

This systematic review provided a comprehensive exploration of the literature regarding collaboration between the members of the “Triangle of Care” regarding mental state changes during an acute mental health admission. The synthesis across the Triangle of Care perspectives provides a unique and holistic understanding of communication across and partnerships within the “Triangle of Care” regarding changes in mental state. The PRISMA guidelines (Tricco 2018) were used to conduct the search and methodological quality was assessed using the JBI Critical Appraisal Tool (Lockwood et al. 2015). The synthesis of results was conducted using the ConQual approach (Munn et al. 2014).

Limitations outside the control of the review include the lack of published literature that provides the family member perspective. Only one study was located, and while a summary of those findings was included, it was not possible to conduct a synthesis of the family perspective using the ConQual approach. A further limitation of this systematic review is that it was unable to include literature that was written in languages other than English, and therefore wider First Nations people and wider cultural perspective on the research question is limited. The study of nurses by Haji Kassim et al. (2021) was conducted in the workplace of the researcher, which may have introduced bias into the findings.

This literature review, however, did not differentiate between the experiences of male and female family members, and this warrants further investigation. Nor did this literature review include research not written in English, and the experiences of these families and mental health services warrant inclusion and exploration.

## 6 Conclusion

Collaboration between all members of the “Triangle of Care” is critical for early identification of mental state changes, and therefore provision of a physically and emotionally safe and recovery‐focused experience for people admitted to acute mental health inpatient units. Personal recovery is promoted by clear and empathic communication of information and the opportunity to ask questions and includes acknowledging and empowering the perspective and contribution of each person. While acknowledging the challenges faced by each member of the triangle, this literature review argues for ongoing organisational support and the attention of each individual nursing staff member to communicate and engage in a strengths‐based manner.

## 7 Relevance for Clinical Practice and Implications for Further Research

This literature review has demonstrated that there is a need for further policy and practice development, and organisational support, to develop a mental health service culture that facilitates communication within the Triangle of Care. This should include training and education of nursing staff in personal recovery and working with families, and provide opportunities for debriefing, clinical supervision and reflective practice discussions. The number of research studies reporting on each group's experience may be reflective of priorities for further research, with those representing the voice of family members (one) the lowest in number and needing further amplification in the future. This impacted on the capacity to conduct a synthesis of studies. It also highlights a particular imperative for future research.

## Funding

This research was supported by the Commonwealth through an Australian Government Research Training Program Scholarship.

## Conflicts of Interest

The authors declare no conflicts of interest.

## Supporting information


**Appendix S1:** Search strategy.
**Appendix S2:** Articles excluded after full text review with reasons.
**Appendix S3:** Critical Appraisal of included studies and dependability score.
**Appendix S4:** Findings (including credibility rating).
**Appendix S5:** thematic analysis conducted by reviewer of included studies.

## Data Availability

Data sharing not applicable to this article as no datasets were generated or analysed during the current study.
