# Supplementary material for: Collaboration Between People Admitted to Acute Mental Health Units, Their Family Members and Nurses in the Detection of Mental State Changes and Recovery: A Qualitative Systematic Review
Source: Int J Ment Health Nurs. 2026 Apr 24;35:e70244. doi: 10.1111/inm.70244 (PMC13108565; doi:10.1111/inm.70244)
Supplement: Supplementary file 1 — Appendix S1: Search strategy. Appendix S2: Articles excluded after full text review with reasons. Appendix S3: Critical Appraisal of included studies and dependability score. Appendix S4: Findings (including credibility rating). Appendix S5: thematic analysis conducted by reviewer of included studies. [file INM-35-0-s001.docx]

Appendix S1 – Search strategy

| Population (P) | Patient, client, consumer, service user, lived/living experience, family, carer, nurse |
| --- | --- |
| Concept (C) | Recovery, engagement, mental illness, collaborate, involvement, deterioration |
| Context (C) | Hospital |

CINAHL/MEDLINE/SCOPUS SEARCH STRATEGY

“mental illness” AND deterior* AND inpatient

“mental illness” AND recovery AND inpatient

“mental illness” AND family AND inpatient

“mental illness” AND nurs* AND inpatient

PSYCHARTICLES SEARCH STRATEGY

Mental health” OR “mental illness OR “mental disorder” OR “psychiatric illness”

Inpatient OR acute OR hospital OR ward OR unit

“family involvement” OR “family engagement” OR family inclusion”

“inpatient psychiatric unit” AND “collaborate” AND “family or families or relatives or parents or siblings or caregiver”

“inpatient treatment” AND collaborat* AND “family or families or relatives or parents or siblings or caregiver”

“mental health care” AND collaborat* AND “family or families or relatives or parents or siblings or caregiver”

“mental illness” AND “family involvement” OR “family engagement” OR family inclusion” AND treatment

“psychiatric patients” AND “family involvement” OR “family engagement” OR family inclusion” AND treatment

“Mental health OR “mental illness OR “mental disorder” OR “psychiatric illness” AND “family engagement” OR family inclusion” AND treatment

“Mental health OR “mental illness OR “mental disorder” OR “psychiatric illness” AND recovery AND “family engagement” OR family inclusion”

EMCARE SEARCH STRATEGY

The following terms were used in various combinations using the BBOLEAN Operators “OR” and “AND”

mental illness

Deteriorat*

Inpatient

recovery

Engag*

collabor*

family

consumer

Nurs*

lived experience

Appendix S2: - Articles excluded after full text review with reasons

Living experience perspective

| Authors | Reason for exclusion |
| --- | --- |
| Adnanes et al. 2020 | Does not directly address research question |
| Ahmad et al. 2016 | Does not directly address research question |
| Badanapurkar et al. 2022 | Does not directly address research question |
| Biran-Ovadia et al. 2023 | Does not directly address research question |
| Cheetham et al. 2018 | Does not directly address research question |
| Cosh et al. 2017 | Wrong participant group – research context was outpatient not inpatient |
| Cutler et al. 2020 | Does not directly address research question – focus is on safety in broad |
| Day & Petalas 2020 | Does not directly address research question |
| DeRuysscher et al. 2020 | Incorrect setting – “residential rehabilitation” as opposed to acute inpatient |
| Duquee al. 2021 | Not written in English |
| Eldal et al. 2019 | Does not directly address the research question – more general experience of hospitalisation rather than addressing engagement or collaboration |
| Favreau et al. 2021 | Does not directly address the research question – focus is on pandemic impact |
| Felton et al. 2018 | Did not directly include perspective of people with living experience |
| Glantz et al. 2023 | Incorrect participant group – nurses not people with living experience |
| Jas 2018 | Does not sufficiently/directly address research question – some participants have forensic background also |
| Jorgensen et al. 2022 | Incorrect context – does not appear to be acute inpatient mental health  Incorrect participant group – professionals rather than people with living experience |
| Kennedy & Fortune 2014 | Does not directly address the research question |
| Lantta et al. 2021 | Incorrect setting – associations rather than acute inpatient setting |
| Lindgren et al. 2018 | Does not directly address research question |
| Molin et al. 2017 | This is a study protocol only, not the primary study – regardless, the participants were nurses not people with living experience |
| Molin et al. 2021 | Does not directly address the research question |
| Mullen et al. 2021 | Does not directly address the research question – it refers to potential involvement as opposed to actual involvement in nursing handover |
| Muddle et al. 2022 | Pre-print – may not have been peer-reviewed |
| O’Keeffe et al. 2016 | Does not directly address the research question |
| Page et al. 2021 | This is not a primary study |
| Pelto-Piri et al. 2019 | Does not directly address the research question |
| Phillips et al. 2021 | Does not directly address the research question |
| Schuster et al. 2020 | Does not address collaboration with nurses but with psychiatrists |
| Schuster et al. 2021 | Does not address collaboration with nurses but with psychiatrists |
| Smith et al. 2021 | Does not appear to include an acute inpatient mental health unit |
| Van de Elde et al. 2024 | No direct responses presented from participants, uncertain if acute inpatient unit |
| Welch et al. 2020 | Not a primary study |

Family perspective

| Authors | Reason for exclusion |
| --- | --- |
| Adams 2020 | unclear if family members are included as “stakeholders” |
| Amsalem et al. 2018 | outpatient clinic and insufficient data from nurses to adequately answer research question |
| Coates 2018 | unclear whether age of “older people” is within the 18-64 group, potentially over 64 |
| Cranwell et al. 2016 | emphasis is not on mental state |
| Filla et al. 2019 | does not directly address research question – focus on social inclusion |
| Law Hale 2017 | not a primary study |
| Haselden et al. 2019 | not a primary study, this is a file review |
| Hsaio et al. 2017 | does not directly address research question |
| Isesolo & Ambikile 2020 | incorrect setting – community not acute inpatient |
| Muddle et al. 2022 | Pre-print – may not have been peer-reviewed |
| Reid et al. 2017 | incorrect setting – specialist mother baby unit not mental health acute inpatient |
| Schuster et al. 2020 | refers to collaboration with psychiatrist not the nurse |
| Vaidya & Patel 2020 | does not directly address research question |
| Varghese et al. 2016 | did not adequately address research question |
| Ward 2014 | participants were parents of young people aged 17-20 – criteria for this study is 18 – 64 |
| Wilson 2023 | did not directly address research question |

Nurse perspective

| Authors | Reason for exclusion |
| --- | --- |
| Berg et al. 2023 | Does not directly address research question |
| Biran-Ovadia et al. 2023 | Does not directly address research question |
| Cleary et al. 2018 | Unclear whether context is acute inpatient unit |
| Cosh et al. 2017 | Not acute inpatient unit |
| Ellacin et al. 2022 | Does not directly address research question |
| Fletcher et al. 2019 | Does not directly address research question, difficult to differentiate perspectives related to acute inpatient context |
| Gabrielson et al. 2016 | Does not directly address research question |
| Grant et al. 2019 | Does not directly address research question |
| Harris & Panozzo 2019 | Not a primary study |
| Hickmott & Raeburn 2020 | Not a primary study – presentation of own living experience |
| Hsiao & Tsai 2015 | Does not directly address research question |
| Jacob et al. 2015 | Incorrect study context – nurses worked in community rather than acute inpatient unit |
| Jorgensen et al. 2021 | Does not directly address research question |
| Jorgensen et al. 2022 | Incorrect study context – unclear if this is acute inpatient context |
| Jorgensen et al. 2024 | Cannot differentiate which nurses work on acute inpatient unit |
| Joseph et al. 2022 | Does not directly address the research question |
| Just et al. 2021 | Can’t differentiate the responses of nurses from those of other professionals |
| Kadir & Fenton 2021 | Not primary research study, this is a service evaluation |
| Kronkvist et al. 2022 | Incorrect context – not an acute mental health inpatient unit |
| McAllister et al. 2021 | Can’t differentiate the responses of nurses from those of other clinicians |
| Milton et al. 2016 | Very limited responses attributable to nurses that work in acute inpatient context |
| Muddle et al. 2022 | Pre-print – may not have been peer-reviewed |
| O’Connor et al. 2018 | Does not directly address the research question |
| Orjasaeter & Almvik 2022 | Does not directly address the research question |
| Santangelo et al. 2018 | Does not directly address the research question |
| Schuster et al. 2021 | Does not include perspectives of nurses – the clinicians are psychiatrists |
| Shue et al. 2022 | Does not directly address the research question |
| Skjaerpe et al. 2022 | Cannot differentiate role of people providing responses adequately |
| Solomon et al. 2021 | Does not directly address the research question |
| Sreeram et al. 2023 | No direct voice/perspective of nurses |
| Vahidi et al 2018 | Does not directly address research question, can’t differentiate responses of nurses |
| Wilson et al. 2023 | Does not directly address the research question |

Appendix S3: Critical Appraisal of included studies and dependability score

| JBI Critical Appraisal Checklist for Qualitative Studies  People with living experience | | | | | | | | | | |  |
| --- | --- | --- | --- | --- | --- | --- | --- | --- | --- | --- | --- |
| Author/Year | Q1 | Q2 | Q3 | Q4 | Q5 | Q6 | Q7 | Q8 | Q9 | Q10 | Dependabilty score |
| Adnanes et al. 2018 | N | Y | Y | Y | Y | U | U | Y | Y | Y | moderate |
| Bradley et al. 2021 | Y | Y | Y | Y | Y | Y | Y | Y | Y | Y | high |
| Eldal et al. 2019 | Y | Y | Y | Y | Y | Y | Y | Y | Y | Y | high |
| Huang et al. 2020 | N | Y | Y | Y | Y | Y | Y | Y | Y | Y | high |
| Isobel et al. 2021 | Y | Y | Y | Y | Y | U | U | Y | Y | Y | moderate |
| Moreno-Poyato et al. 2020 | N | Y | Y | Y | Y | N | Y | Y | Y | Y | high |
| Olasoji et al. 2018 | N | Y | Y | Y | Y | U | U | Y | Y | Y | moderate |
| Olasoji et al. 2020 | N | Y | Y | Y | Y | N | N | Y | Y | Y | moderate |
| Ould Brahim  et al. 2020 | Y | Y | Y | Y | Y | Y | N | Y | Y | Y | high |
| Schon 2013 | Y | Y | Y | Y | Y | N | N | Y | Y | Y | moderate |
| Van de Velde  et al. 2021 | Y | Y | Y | Y | Y | Y | Y | Y | Y | Y | high |
| Waldemar et al. 2018 | Y | Y | Y | Y | Y | N | N | Y | Y | Y | moderate |
| Waldemar et al. 2019 | Y | Y | Y | Y | Y | N | N | Y | Y | Y | moderate |
| Wilson et al. 2023 | Y | Y | Y | Y | Y | N | N | Y | Y | Y | moderate |
| Wyder et al. 2015 | Y | Y | Y | Y | Y | Y | N | Y | Y | Y | high |
| Wyder et al. 2016 | Y | Y | Y | Y | Y | Y | Y | Y | Y | Y | high |
| Wyder et al. 2018 | Y | Y | Y | Y | Y | Y | Y | Y | Y | Y | High |

| Family members | | | | | | | | | | | |
| --- | --- | --- | --- | --- | --- | --- | --- | --- | --- | --- | --- |
| Schaffer 2021 | U | Y | Y | Y | Y | Y | N | Y | Y | Y | high |
| Nurses | | | | | | | | | | | |
| Chambers et al. 2015 | N | U | U | U | U | N | N | Y | U | Y | low |
| Cheetham et al. 2018 | Y | Y | Y | Y | Y | Y | N | Y | U | Y | high |
| Cleary et al. 2013 | U | Y | Y | Y | Y | N | Y  (minimal) | Y | Y | Y | high |
| Digby et al. 2020 | Y | Y | Y | Y | Y | U | N | Y | Y | Y | moderate |
| Foster & Isobel 2018 | Y | Y | Y | Y | Y | N | N | Y | Y | Y | moderate |
| Gwinner & Ward 2015 | U | U | Y | Y | Y | N | N | Y | U | Y | low |
| Haji Kassim et al. 2021 | U | U | Y | Y | Y | Y | N | Y | Y | Y | moderate |
| Hristodoulidis et al. 2020 | Y | Y | Y | Y | Y | Y | N | Y | Y | Y | high |
| Jorgensen et al. 2020 | Y | Y | Y | Y | Y | N | N | Y | Y | Y | moderate |
| Lim et al. 2019 | Y | Y | Y | Y | Y | Y | Y | Y | Y | Y | high |
| McKenna et al. 2014 | U | Y | Y | Y | Y | N | N | Y | Y | Y | moderate |
| Tucker et al. 2020 | Y | Y | Y | Y | Y | Y | Y | Y | Y | Y | high |
| Waldemar et al. 2019 | Y | Y | Y | Y | Y | Y | Y | Y | Y | Y | high |

JBI Critical Appraisal Checklist for Qualitative Research (from Lockwood et al. 2015)

1. Is there congruity between the stated philosophical perspective and the research methodology?

2. Is there congruity between the research methodology and the research question or objectives?

3. Is there congruity between the research methodology and the methods used to collect data?

4. Is there congruity between the research methodology and the representation and analysis of data?

5. Is there congruity between the research methodology and the interpretation of results?

6. Is there a statement locating the researcher culturally or theoretically?

7. Is the influence of the researcher on the research, and vice-versa, addressed?

8. Are participants, and their voices, adequately represented?

9. Is the research ethical according to current criteria or, for recent studies, and is there evidence of ethical approval by an appropriate

body?

10. Do the conclusions drawn in the research report flow from the analysis, or interpretation, of the data?

Appendix S4: Findings (including credibility rating)

Living experience group

| Study 1: Adnanes et al. 2018. Mental health service users' experiences of psychiatric re-hospitalisation - an explorative focus group study in six European countries |
| --- |
| Findings: Commonalities emerged across all six countries involved in the study. (U)  Four themes:  (1) re-hospitalisation as less traumatising than the first hospitalisation (E) |
| Illustration: “I must say that now – the second time around was better, although the hospitalisation itself, the way they held me in was terrible. I must say that I was still scared because of it for a while. One day I was at a shopping mall, after I was released from the hospital, when I came home. There was a group pf people, and I was immediately afraid that they would attack me…” (S 1/3) p. 519 |
| (2) re-hospitalisation as a necessity and a relief (U) |
| Illustration: “Yes, it was a relief that I went, like I was going to a vacation. So, I can rest my brain and pull myself together and then forward again” (S 2/14) p. 519 |
| (3) re-hospitalisation by default without progress (U) |
| Illustration: “A few participants even blamed mental health services themselves of sustaining mental health problems, illustrated by the following: “[..] now it’s like people come [to hospital] again and again, and why do people come in again and again and again? Well, people come in again and again and again because psychiatry “chronicises” instead of helping people regain their health.” (F/2) p. 519 |
| (4) re-hospitalisation as part of the recovery-process. (U) |
| Illustration: “In my opinion, you have to think that it [re-hospitalisation] is a part of life. Admission to hospital is natural; it is not a big deal. If you have to go to the hospital to have your appendix removed, not many people question that, right? But, if you have [a chronic illness], it is partly a defeat, but partly you should also think that it is natural.” (N/1). p. 520 |

| Study 2: Bradley et al. 2021. It's a little bit like prison, but not that much: Aboriginal women's experiences of an acute mental health inpatient unit |
| --- |
| 5 themes:  1: Social context – we’ve come from a lot of stress (U) |
| Illustration: “Everything was about him. He want to go to the pub, poker machine … he’d wait for my payday and beg me to come back – take my money again” (Greta IP) p. 919 |
| 2: Connections – feeling alone and feeling abandoned (U) |
| Illustration: “Generations and to substance abuse, illness, and death. Some inpatient participants described a deep, draining sadness stemming from isolation from kin. Sometimes when they don’t call me on the phone … sometime I go in the bed and cry, cry, cry – sit alone on the chair, crying like a baby, trying to rock myself (Olivia, IP) p. 920 |
| 3. Control – I’m always locked in (U) |
| Illustration: “Inpatient participants resented loss of freedom to access the outside world and to collaborate in decision-making about leave with family: Just [staff] to accept ... if they [patients] want to go out for themselves and explore and see their future and then come back. ... I just don't want to stay here because I'm always locked in. Not going out, to see and explore, that's why (Janet, IP) pp. 921-2 |
| 4: Caring – be there for us. (U) |
| Illustration: Nurses were often perceived to avoid opportunities to engage with women wishing to share thoughts and plans: Sometimes - sometimes they don’t want to be interrupted: that’s what they say, the nurses. ... And that’s the reason why some [patients] . . . just want to stay inside their room, and sometimes want to sit quietly and not be telling the truth, who they want to be - that’s the reason (Janet, IP). (p. 922) |
| 5: Communication – there’s a lot if information I can’t understand (U) |
| Illustration: “Make sure the nurses and social workers and AMHWs are letting us know that they’re there when we need to talk to them. And y’know just letting us know when we come in – and even keep on telling us - “we’re here” (Teresa, IP)” p. 924 |

| Study 3: Eldal et al. 2019. Contradictory experiences of safety and shame in inpatient mental health practice – a qualitative study |
| --- |
| Findings: 2 themes – “complex duality of patient experiences” (p. 795)  1. a place where it is alright to be vulnerable (U) |
| Illustration: “Ehhh... Compared to how it is at home I think this is just so very safe, having people around me, and being able to withdraw if I want to, and having some routines ....” (Participant 8, p. 794-5) |
| Illustration: “It’s that you’re … or I’m scared that … that I’ll just stay on here in a way, and over time .. and scared that I will lose touch with family, friends … (long silence) work … (long silence. …” (Participant 7, male) p. 795 |
| 2. the burden of everyday stigma and signs of depersonalisation. (U) |
| Illustration: “there are staff who just make things worse…” (Participant 8, p. 795) |
| Illustration: You’re initially allowed to feel down for a little while when you’re hospitalized – but then it’s sort of expected that you get over it. Pull yourself together. Go out there and sit! (Participant 13 p. 795) |
| Implications for practice: “stakeholders must have flexible attitudes, be open-minded and dare to ask the patients of their needs. Recovery-oriented practices and open dialogue-based approaches may be measures that can be used by staff to develop more reflective practice “ p. 798 |

| Study 4: Huang et al. 2020. I am the person who knows myself best: Perception on shared decision-making among hospitalized people diagnosed with schizophrenia in China |
| --- |
| Findings: Three main themes   1. Having a positive attitude – wanting my voice to be heard, needing my family to be involved, preferring the psychiatrist to decide. (U) |
| Illustration: “When we are fully involved in the whole process of treatment, we could have a personal understanding and really know where we are, which is an encouragement and can enhance self-confidence.” (Participant 5 p. 849) |
| Illustration: “…It is not necessary to involve nurses in decision-making. They only take care of the patient and do not know how to prescribe medications and treat illness. They can only draw blood and administer medication according to the doctor’s orders (P 11) p. 850 |
| 1. Feeling excluded – having limited financial capacity, lacking interactive communication, too few psychiatrists, being unsatisfied with the informed consent process. (U) |
| Illustration: “…”I was changed to amisulpride but they didn’t tell me the potential side-effects. I am suffering from the side-effects now but they never asked my feelings. From another point of view it was not perfect and inhumane” (P12). p. 851 |
| 1. Self-motivation in decision-making. – easing the burden on the family, actively collecting health information. (U) |
| Illustration:: “Because I am the pillar of the family. If I were worn down, the sky would fall, so I want to cooperate with the doctor. I should be responsible for my family, I still have filial responsibility to my parents. “ (Participant 7, p. 852). |

| Study 5: Isobel et al. 2021. 'What would a trauma-informed mental health service look like?' Perspectives of people who access services |
| --- |
| Findings: Trauma-informed care requires   1. Being aware of trauma - Need for mental health services to discuss and consider trauma experiences in care, reflected in the way services make people feel (U) |
| Illustration: “I don’t want to sit and rehash trauma but I want them to discuss it and help me pick out links” p.498 |
| 1. Collaborating in care. (U) |
| Illustration: “Instead of writing a note about someone where you type up the mental state assessment or talk about. . . ‘Bob was making good eye contact and wearing a blue sweater but he seemed a bit stinky and I wonder if he’s off his meds’. . . [ask me] what feels important to say, what would you like in the notes. . .so people have control about what’s being said about them in their absence’. “ p. 498 |
| 1. Building trust. (E) |
| Illustration: “I think I have more trust in an organisation staffed with people with lived experience…you know how terrible it is to feel powerless …” p. 498-9 |
| 1. Creating safety ( E ) |
| Illustration: ‘There’s literally nothing to do. How can you make some-one get better when you’re sitting them in a box all day long? Especially when they’re in seclusion, you’re going to send them crazy. That sends anyone crazy’. p. 499 |
| 1. Delivering a diversity of models. (Unsupported) |
| Illustration: “The language is so alien and clinical and distant”. p. 499 |
| 1. Staff practising with consistency and continuity. (U) |
| Illustration: “sometimes there's one nurse or one person you come into contact with that really can support you” p. 500 |

| Study 6: Moreno-Poyato et al. 2021. A qualitative study exploring the patients' perspective from the 'Reserved Therapeutic Space' nursing intervention in acute mental health units |
| --- |
| Findings: 3 topics covered   1. The meaning of a space in order to establish a therapeutic relationship (U) |
| Illustration: “The space doesn’t matter if there is a dialogue. It’s not so much where so much as how” (FG3P6) p. 786 |
| 1. The procedure to implement the Reserved Therapeutic Space (U) |
| Illustration: “I would have liked to receive a welcome from the first moment and an introduction by the nurse ... an open door for me to ask questions and talk to her FG1P4 The nurse has to generate trust and, to do so, she has to convey confidence, be affectionate, give patients the option to express themselves and talk about their concerns” (FG3P6) p. 796 |
| 1. The difficulties to overcome in order to establish the Reserved Therapeutic Space. (U) |
| Illustration: “We assume that involuntary admission is a violation of rights ... it is difficult to maintain a therapeutic relationship . . “ (FG1P2) p. 787 |

| Study 7: Olasoji et al. 2018. Views of mental health consumers about being involved in nursing handover on acute inpatient units |
| --- |
| Findings: Handover is about communication and care planning, people with living experience want regular information, not ad hoc  2 themes:  1. Behind closed doors – it is about us, knowing their thoughts (U) |
| Illustration: “It’s important that we can contribute, because we are the subject” (Participant 7) p. 750 |
| 2. Being involved – clarifying issues, setting expectations, when and how (U) |
| Illustration: “The only thing would be is if you felt uncomfortable during handover. If it took place say in your room, and it’s a shared room, I guess during handover you wouldn’t want any other clients to hear what the nurses are saying: you definitely wouldn’t want (that). “ (participant 3) p. 752 |

| Study 8: Olasoji et al. 2020. The benefits of consumer involvement in nursing handover on acute inpatient unit: Post-implementation views |
| --- |
| Findings: 3 Themes   1. Knowing who (U) |
| Illustration: “Yeah, that way we get to exchange information, they know who I am, and I know who they are. (Participant 2)”p. 789 |
| 1. Shared decision-making (U) |
| Illustration: “A few days ago, I was having a really bad day and they[morning and afternoon nurses] came and introduced me to my afternoon nurse and said did I have any me to my afternoon nurse and said did I have any problems and I was like I’m feeling really down, is there anything you guys can do? They went and got me some medication and stuff so they act upon it. My afternoon nurse said I’ll come and I will talk to you later and she stuck by that and actually came and got me and sat me down for a chat later on.” (Participant 2) p. 790 |
| 1. Having time and space ( E ) |
| Illustration: That’s the part that I’m not sure of. So, it feels some- times your privacy is respected and sometimes it feels as if it’s not. (Participant 4) p. 791 |

| Study 9: Ould-Brahim et al. 2020. Understanding Helpful Nursing Care From the Perspective of Mental Health Inpatients With a Dual Diagnosis: A Qualitative Descriptive Study |
| --- |
| Participants were either admitted to an acute psychosis, mood disorder or psychosocial rehabilitation inpatient unit – it is unclear from the paper which participants were admitted to which unit  Findings: three main themes: - the majority of helpful nursing practices pertained to relational care. ( E )   1. promoting health in everyday living eg: physical health |
| Illustration: “You see that there are health professionals who … try to fix things with family … it’s reassuring, it’s a good feeling, it’s like love … you have help around you to make it out, to do something with your life. (S26, F, 30). p. 255 |
| 1. managing mental illness in tandem with substance use |
| Illustration: Everyone should get some kind of education before they leave [the hospital] about their problem . . . not just given drugs like . . . about the diagnosis about . . . what’s the best way of dealing with it. (J20, M, 25) p. 255 |
| 1. building a therapeutic relationship eg being treated humanely. (U) |
| Illustration: “Another participant advised. nurses to: “talk about how [patients] feel, be open to them, have a good time with them, and spend time with them” (N30, F, 23). pp.255-6 |

| Study 10: Schon 2013. Recovery in involuntary psychiatric care: Is there a gender difference? |
| --- |
| Findings: Recovery is an individual process with some similarities and differences. There are gender differences in how coercion and compulsory care are experienced and described. Four dimensions were described  1. ambivalence. ( E ) |
| Illustration: “The positive factors included staff members “whom you trusted” or who were “knowledgeable”. p. 423 |
| 2. experiences of oppression. (U) |
| Illustration: “Psychosis is very much about oppression. Self-repression or oppression from others. And then what happens when you enter [inpatient care] is new oppression, and it becomes the new constraint” (woman 1). p. 423 |
| 3. absence of treatment (U) |
| Illustration: I think the best thing that could happen to psychiatry would be if they said we cannot do much, but maybe we can help a little. We can offer a bed, food, and medicine. But if you realise that, you can spare the patients immense disappointment. (Woman 4) p. 424 |
| 4. medication. ( E) |
| Illustration: ‘The two women with bipolar disorders wanted to continue their medication but also wanted the dosage to be “as low as possible’. p. 425 |

| Study 11: Van de Velde et al. 2021. Nursing handover involving consumers on inpatient mental healthcare units: A qualitative exploration of the consumers' perspective |
| --- |
| Findings: “being able to immediately connect with nurses is an important condition for a successful recovery process”  “ nursing handover involving consumers, consumers experience they have an opportunity to take more control over their care process.”  Both quotes p. 1723   1. themes 2. The first moments at the inpatient mental health unit. (U) |
| Illustration: “The difference was that in the beginning I was not sure exactly they were doing. What should I expect? But they did take their time to explain that briefly. And after a week or a week and a half I started to understand it very well and I started to feel the need for that moment” (Consumer 11). p. 1719 |
| 1. The nurse as an ally. (U) |
| Illustration: While here they come to your bed and they say what they know and you can possibly add to that, because they always ask, “Do you have anything to add?” and I think that is very important that you know “They know this, can I add anything more? Do I have anything else on my mind that I want to say?” That’s important. Consumer 3 (p.1720). |
| 1. Informing each other. ( E ) |
| Illustration:  “In your room they are in your personal space, in fact. When you are at the desk, they are always busy, so it sometimes is like “Do you have a minute?”. I think it is more productive that they come to your room because then they have their full attention. Otherwise, if you ask questions between their papers and telephones ... Yes, there just arrived three people before me so the chance that they will remember my question half an hour later is perhaps small. Yes, you always think that your question is the most important question, but that is not the case. So, that they come into your personal space, of course they then make time for it. They write down everything immediately, they ask questions, they call immediately if necessary. (Consumer 10)” p. 1721 |

| Study 12: Waldemar et al 2018. Recovery orientation in mental health inpatient settings: Inpatient experiences? |
| --- |
| Findings: 6 themes   1. being accepted and protected. (U) |
| Illustration: “It kind of stabilizes me because I don’t have to keep myself together. Like, I always put up a façade to my friends, I don’t want them to see how bad I feel, and in here I can drop that façade. In here I’m allowed to be sad – or actually I allow myself to be sad.” (Participant 14). p. 1181 |
| 1. having company yet longing for dialogue |
| Illustration: “I feel like they [health professionals] take care of me. There’s a safety net out here. If I’m feeling really bad, I have someone to go to. In that aspect, I feel like it’s helpful to be here. If I’m at home and feel bad, I feel like I’m all alone in the world. But here there are people I can go and talk to. (Participant 5)”p. 1181 |
| 1. In the dark, confused, and uninformed |
| Illustration: I’m guessing that there is a plan... also when I think about the fact that they told me that there isn’t any progress in the current treatment and that they want to try something else, then they must have some sort of plan for where I should be by now... and that I am not there. (Participant 1)“ p. 1182 |
| 1. being observed and assessed |
| Illustration: “Everything you tell your primary nurse goes into the report. . .. as a patient, it would be nice if not every- thing you say gets reported. When I became aware that they wrote down everything, I said I didn’t feel like talking to them anymore. (Participant 9)”p. 1183 |
| 1. limited choice and influence |
| Illustration: “I don’t feel that I have that much of an influence. I feel like I’ve been told, ‘This is what you suffer from and this is what will help you”, and that’s actually okay …” (Participant 13) p. 1183 |
| 1. Treatment centred on medicine |
| Illustration: Of course, this is real treatment, but it’s mostly medical treatment. Focusing on how I am as a person comes later on, when I start processing things. In that way, this is more like an introduction to psychiatric treatment. Not that this isn’t real treatment, it’s just that its more of a medical focus (Participant 6)”p. 1183 |

| Study 13: Waldemar et al. 2019. Recovery-oriented practice: Participant observations of the interactions between patients and health professionals in mental health inpatient settings |
| --- |
| Findings: One overarching theme – “as-if collaborating” – “shallow or artificial interactions, decisions made beforehand or subjugating more pertinent demands than patient’s needs (p. 329)  Illustration: "I ask patient Eric how his preferences and wishes informs the treatment and care he receives on the ward. He tells me “well, they ask me about it, that’s for sure – what I want and think about things. But I’m not sure what that information is used for in the planning. I feel like they listen to me when they assess whether I’m ready for transfer, I think that it’s probably mainly there my inputs mean something.” “ p. 324 . It is unclear who the “they” is that Eric refers to – a Doctor? Nurse?   1. subthemes 2. negotiating on limited grounds. ( E ) |
| Illustration: “A male patient goes to the closed office door and knocks. Nurse Jane opens the door and the patient says to her that he’ll agree to take Antabuse, and wants to take the next bus home. The nurse tells him that she’ll let the doctor know. The patient then says that he doesn’t really want to take it, but that he’ll do it in order to go home, because he feels uncomfortable here” p. 324 |
| 1. competing demands. ( E ) |
| Illustration; “Patient Theo and the peer support worker are talking about a hospital they’ve both been to. A nurse comes and tells Theo that he must go see the psychiatrist and he leaves with her. Twenty minutes later Theo returns to the living room and declares that he has to say goodbye now because he is being “kicked out’ …”. p. 325 |
| 1. inconsistent guidance and postponed decisions. ( E ) |
| Illustration: “Two nurses and nurse assistant are in the staff office. Patient Sarah comes to the door and says ‘why is Sofie my primary nurse today, instead of Anders? It’s so confusing!” The nurse tells her that’s it’s because of a system they have so she can’t rely on having the same primary nurse every day. Sara sighs and says, “ok well, I guess I just have to deal with even more people then.” p. 325 |
| 1. control and condescending communication. (unsupported) |
| Illustration: no quotes attributable to nurses – only nurse assistants |

| Study 14: Wilson et al. 2023. Promoting mental health recovery by design: Physical, procedural, and relational security in the context of the mental health built environment |
| --- |
| Findings: 3 themes  1. Rooms should be designed to promote physical security (U) |
| Illustration: “all rooms to have an ensuite would be nice, yes … Even get up in the middle of the night to go to the toilet you’re kind of self-conscious .. you’ve got to put clothes on or whatever just to get to the toilet and come back again” (SU) p. 154 |
| 2. Purposeful planning should be supportive of interactions between users and systems to promote relational security |
| Illustration: “you can’t get anyone to notice you (when waiting for permission)” (SU) p. 155 |
| 3.. Service integrity should promote procedural security |
| Illustration: no quotes attributable to people with living experience |

| Study 15: Wyder et al. 2015. Therapeutic relationships and involuntary treatment orders: Service users' interactions with health-care professionals on the ward |
| --- |
| Findings: Range of overall positive, negative and mixed experiences – staff behaviours and attitudes shaped their experience  Procedural injustices – agency and control were critical  What are good relationships? Feeling connected, Relationships based on partnerships |
| 1. Staff potential to impact on ITO and hospital experience |
| Illustration: Without (Nurse A) and (Nurse B), I don’t think I would have managed it in here. We just clicked and I can tell them anything. They don’t leave me there to sit and they just chat to me. (Dun et al.) p. 185 |
| 1. What are good relationships? |
| Illustration: Many staff members treat you like a case or a number. . . . All they do is increase your medication and they do not look at the full package of the person. They just think that the medication is the answer to everything. (Evelyn) p. 187 |

| Study 16: Wyder et al. 2016. The importance of safety, agency and control during involuntary mental health admissions |
| --- |
| Findings: Overall experience of the ITO   - Protected them from harm - ITO is an intrusion on my liberties |
| 1. Factors affecting sense of agency – having a safe place to reflect on their experience |
| Illustration: Ryan. “I had some time to think about things and put things in perspective, […] I feel safe. I feel that I can talk to people just any time and just stuff like that about my problems and I feel connections with people.” p. 340 |
| 1. Understanding their ITO conditions and ward expectations |
| Illustration: Isabella: ‘‘Absolutely, finding out who’s doing what [is difficult] [. . .] Even writing the nurses’ names and stuff on the board. It is not always done. We’re constantly asking, who’s my nurse? When we are asking for our medication, they are either too busy, on a break or it is changeover time.’’ p. 341 |
| 1. Having input into their treatment |
| Illustration: Ellie: ‘‘I get different injections and I try to explain that they are giving me too many but I get dismissed and they don’t listen. It makes me feel upset and like a guinea pig.’’ Amelia ‘‘I like the psychiatrist as he is involved and listens. He makes changes to my medication when I ask him. I like his thinking about medication and mental illness. He is always asking me how are you coping and how I can manage. He really listens.’’ p. 341 |

| Study 17: Wyder et al. 2018. Our Sunshine place: a collective narrative and reflection on the experiences of a mental health crisis leading to an admission to a psychiatric inpatient unit |
| --- |
| Findings: Overarching experiences   1. Making sense of what happened leading up to the admission. ( E ) |
| Illustration: “The doctor is technically competent but just so impersonal…. I want to feel valued and encouraged. I want the staff to be supportive and aware of me” p. 1243 |
| 1. Potential impact of the illness on the future. (U) |
| Illustration: “I want to feel hopeful about my future and that I can recover from the mess – the mess my mental illness left in my life. “ p. 1243 |
| Factors that facilitated or hindered recovery   1. A place of safety |
| Illustration: “Everything just felt threatening and unsafe. I wasn’t even allowed to be scared. Can’t they see how alien this place is? How alien I feel? [...] I know I am here to be safe but I am just so scared. This place does not feel safe. [...] “ p. 1244 |
| 1. Feeling connected |
| Illustration: “I like listening to the nurse’s conversations about their lives and work. It is comforting to know that there is a life outside these walls …”. p. 1244 |
| 1. Autonomy and control |
| Illustration: “Even after being injected, nobody spoke to me about this. Why aren’t we given some choices about the drugs we take? Why don’t they ask me? Talk to me? I want to know why I am on the drugs! “ p. 1245 |

family perspective

| Study 1: Schaffer 2021. Family perspectives of healthcare for relatives living with a mental illness |
| --- |
| Findings: Each group identified some positive and negative experiences, however differences were evident according to role. Helpful responses eg: information, empathy (depended on group/role). Theme of helpful responses for parents and partners/spouses and medication issues identified for both partner/spouse and adult children groups. Parents and partners had largest burden for finding resources and struggled the most with confidentiality issues. Adult children were less likely to experience positive responses from the mental health system.  Themes of experience (p 1550)  Parents (U)   - Reliance on service systems - Confidentiality rules as a barrier - Mental healthcare as a rollercoaster - Helpful responses - Presence of provider/staff empathy |
| Illustration: “She explained, “my son went to the hospital. I’m balling uncontrollably. I’m looking for resources. I go to a support group. I’m learning more. And then my son hates me and we can’t get any information from the hospital”. p. 1550 |
| Partners/spouses. (U)   - Concerns about medications - Communication challenges - Unable to get attention of staff - Helpful responses |
| Illustration: “A woman who was trying to convince staff that her partner was not ready for discharge from a mental health unit, resorted to recording a middle‐ of‐the‐night phone call from him to provide evidence of concerning symptoms to the psychiatrist. I said, “I want you to hear this and listen. This doesn't sound like the person you're talking about.” When he [provider] heard it, he totally changed his tune. But I had to convince him and I had to have that proof because I don't know if [my partner] had him snowed or what, but they were going to send him home. With that being recorded and having that doctor listen to it, what more could he say. He's not okay” p1551-1552 |
| Siblings (U)   - Taking on responsibility to get help - Gratitude for resources |
| Illustration: “I learned if he’s not threatening his life or someone else’s, you can’t get him into the hospital. It’s been a lot of taking this all on myself and feeling like I had to be a martyr” p. 1552 |
| Adult children (U)   - Impact of hospitalizations and medications - Cannot talk about it - Desire for more attention to family members - Healthcare system inadequacies |
| Illustration: “A daughter and her family had to quickly come up with a plan when the hospital unexpectedly discharged her mother. She lamented about the challenges in mental health treatment, “Mental illness is so hard. You can't scan the brain. Only if you could—oh here's the magic mix that you need.” p 1553 |

nurse perspective

| Study 1: Chambers et al. 2015. Managing and caring for distressed and disturbed service users: the thoughts and feelings experienced by a sample of English mental health nurses |
| --- |
| Findings: Mainly negative feelings eg: fear anxiety and vulnerability Conflict between benevolence and malevolence  Nurses ready and open to try different care and management approaches Use therapeutic relationship to help de-escalate    Understand triggers and antecedents. Interaction and communication should be meaningful,  sincere and positive  “Overall, this study illuminates that the English nurses’ experience of using coercion in psychiatric care is a very complex and delicate matter that is complicated by the tradition of coercion in psychiatric care, the nature of mental disorders and relationships among nurses, patients and other health care professionals.” p. 294  3 overall themes  1. emotional and cognitive dissonance. (U) |
| Illustration: *“*I’ve seen staff shy away or don’t want to get involved because they feel either they will be hit by the patient or something might go wrong . . . most people they are not comfortable doing it and they will avoid it if necessary. . . . You are in a very vulnerable position. . . . I actually froze because I was quite petrified . . . feeling quite anxious.” p. 292 |
| 2. therapeutic engagement. (U) |
| Illustration: “Through communication you learn what the problem is, and also about listening, to what the patient wants and you have to pick up, the frustration or why he is feeling the way he is . . . It’s about knowing your patient. . . . It’s kind of about respect and normal interactions.” p. 292 |
| 3. organizational management and support. (U) |
| Illustration: “There should be room for de-escalation support on the day of the attack. The only time I’ve seen de-briefing being done is after a restraint and that’s because it’s paperwork, . . . we make sure we do debriefing with the patient, I can’t really remember there being formal debriefs” p. 293 |

| Study 2: Cheetham et al. 2018. ‘I can see it and I can feel it, but I can't put my finger on it’: A Foucauldian discourse analysis of experiences of relating on psychiatric inpatient units |
| --- |
| Findings: 3 discourses  1.The most dominant discourse was the medical-technical-legal discourse (U) |
| Illustration: Aardash “It’s my experience, my clinical knowledge on the symptoms, manifestations, the knowledge of the patient’s diagnosis, their presentations, their treatment plan, what is in their PRN medication list, and you know your general use of communication on a daily basis, problem solving approach, solution focused techniques, a bit of CBT [. . .] you know your patient, you know who you’re working with you know what will work for them, it’s tested and tried”. (L224-238) (p. 322). |
| 2.Counter discourse of Ordinary humane relating – experiences of ordinary care, compassion and concern (U)  Tensions/dilemmas exist between ordinary acts and the environment they occur in could be hard to verbalise |
| Illustration: “it’s nice to know that you’ve connected with somebody, then you can sit down and like have a conversation with them and you know that they’ll talk to you and open up to you. (L317-321) “ p. 325 |
| 3.Collaborative exploration – the value of purposefully therapeutic encounters (U) |
| Illustration: Marika “some of the staff would be a bit stand offish or not as talkative because they’re not sure how to take the patient, whereas I went and spoke and was like, oh where are you from, what brings you in here, how come you feel like that and even, just staying in a simple conversation and then after that her mood changed [. . .] I think as well it helped her to not have her guard up on the ward. (L194- 199; 225-227) |

| Study 3: Cleary et al. 2013. Mental health nurses' views of recovery within an acute setting |
| --- |
| Findings: Nurses had different opinions of recovery  “Nurses in our study did not clearly incorporate empowerment, social inclusion, peer support, or public education about mental illness in their recovery definition ”… “which may reflect their role as acute inpatient nurses and the notion of recovery being regarded as an outcome “ p. 209  These findings draw attention to the significant overlap of recovery principles and processes with those of humanistic and holistic care provided by nurses in acute care settings” p. 209  “However, given the short length of stay in hospital, most interviewees considered their employers’ claim to recovery-orientation more as rhetoric than an appropriately resourced, coordinated, and integrated program” p. 209  Education needed on the practical ways of contributing to recovery  “Realistically, improving recovery-oriented services involves system-wide changes involving restructuring, working in partnership with providers, comprehensive training, and ensuring seamless coordination across services, particularly between hospitals and the community” p. 210  “Need to challenge and clarify some values of nurses and also enact cross disciplinary collaboration” p. 210   1. overlapping themes 2. Perception of recovery. (U)   most prominent idea – holism  a second group of responses reflected a medical view  smallest group of responses related to being able to manage activities of daily living  more than half did not see recovery imbedded in structures or functioning |
| Illustration: ‘recovery means getting well and being able to return to a pre-illness functioning level on all aspects; physical, emotional, psychological, mental and social’ p. 208 |
| 1. Humanism (U)   This refers to aspects of “humanistic interpersonal nursing” (p. 208) |
| Illustration: “Examples include interacting with patients as whole human beings, developing a therapeutic relationship, communicating well, ‘counselling’, being kind, offering encouragement and positive reinforcement, ‘listening’, discussing options, being positive, hopeful, and optimistic, and ‘talking about the future’.” p. 208 |
| 1. Practical realities. (unsupported) |
| Illustration: In response to asking about nursing support of consumers’ recovery, five topics were repeatedly mentioned. In order of frequency these were: (i) medication; (ii) education; (iii) goals and discharge planning; (iv) interpersonal relationships; and (v) social and practical aspects of daily living. For example, one nurse’s unit supported recovery ‘by setting up groups (and) stabilizing mental illness and medications while in hospital, we help them solve their financial or relationship issues; we give pharmacological education, we help them to understand mental illness and set up goals, and help them start the journey of recovery’. Attitude is emphasized, too: ‘the expectation of recovery aids the patient, as it is communicated to them from us and they feel that recovery is possible’. p. 208 |

| Study 4: Digby et al. 2020. Implementing a Psychiatric Behaviours of Concern emergency team in an acute inpatient psychiatry unit: Staff perspectives |
| --- |
| Findings: 4 themes   1. identifying deterioration - identifying early warning signs, knowing/not knowing the patient (U) |
| Illustration: “The signs of impending problematic behaviour were not the same for each patient. The ability to see and interpret changes in patient behaviour and intervene in the early stages were core skills in treating and managing aggression” “Ability to read it coming on...Change to the person that they’ve been. An increase in their rapid speech. . .Pacing the ward. . .their loudness . . . threatening. . ., you know that’s escalating towards physical (Nurse group) p. 891 |
| 1. Responding to behaviours of concern (U) |
| Illustration: “The nurses reported feeling reassured that the PsyBOC team were available to help when they had been unsuccessful in resolving a situation. we had tried everything we could think of and it worked well because some of the people who attended the scene knew this particular patient quite well and were able to spend some 1:1 time with him and yeah, managed to de-escalate him, yeah but him being settled for a period after was a different story, he stayed settled for about half an hour. (Nurse group)” p. 893 |
| 1. Staff reactions to Psy-BOC. (U) |
| Illustration: “I feel like I just need to be on the in because I am the person that’s going to be looking after that patient … there is safety in numbers, but when they leave am I really that safe? … it could just be a placebo, cushioning effect … (Nurse group) p. 895 I think what’s really important is that the junior people to have the opportunity to see good de-escalation in action and in process...I think we miss that education frequently... it’s a missed opportunity, and I think in terms of when someone can de-escalate and de-escalate somebody, there needs to be another clinician in the room so they can just hear how that is effectively done. . . and then have somebody explain [it] to them. (Nurse group) p. 895 |
| 1. Barriers to Psy-BOC (U) |
| Illustration: “… it’s someone talking to the patient that doesn’t even know the patient … and the people that are working in there with the patient for the past 5 days feel undermined … what are you even talking about, that’s not going to work (Nurse group) p. 896 |

| Study 5: Foster & Isobel 2018. Towards relational recovery: Nurses’ practices with consumers and families with dependent children in mental health inpatient units |
| --- |
| Findings:  1. balancing risk and safety - The strongest emphasis was on risk aversion and safety management (U) |
| Illustration: “A lot of what we do is risk management...the highest risk group would be the most vulnerable, so if you can stop them attending the unit, it’s one problem solved. “ (Nurse 20) (p. 730) |
| 2. facilitating family connections. (U) |
| Illustration: “I think it works well because we want to work together with our clients so we can give them the best possible care and...they obviously want to be with their families and have some time with their families and...it gives them time with their family. “ (Nurse 10) p. 730 |
| 3. incorporating family rooms within care provision. (U) |
| Illustration: “I think there’s a lack of policy around some of this....There’s a lot of descriptive things about involving families and all these principles, but it’s not translated well into policy and into practice on a unit. So I find units will evolve their own culture around how they manage that” (Nurse 20) p. 731 |

| Study 6: Gwinner & Ward 2014: Storytelling; safeguarding; treatment; and responsibility; attributes of recovery in Psychiatric Intensive Care Units |
| --- |
| Four attributes of recovery specific to a PICU environment   1. storytelling – engagement/interactions between nurse and patient are used to discern subjective experience. (U) |
| Illustration: “break that ice initially, then you get a better rapport and a better working relationship with that person in a PICU”. p. 109 |
| 1. safeguarding – relate to safety and care of patient eg: milieu, care plans, lack of collaboration. (U) |
| Illustration: “The trouble you get with patients in a locked area is the amount of rules you have. So I think the minimal amount of rules but you do have everyone sticks to. “ p. 110 |
| 1. Treatment – an underdeveloped aspect of recovery practice. ( E ) |
| Illustration: “We have got weighted blankets and stuff like that but umm and stress balls and … but we have to be with them when you do stuff like that.”  p. 111 |
| 1. Responsibility – accentuates the balance of dignity, respect and human rights with risk and decision-making. (U) |
| Illustration: “It is also part of the person and whether they really want to recover as well, and that’s where you could establish support and try to ... find out some of the client’s issues and look at the objectives of what this person really wants and where they really want to go with it.”  p. 112 |

| Study 7: Haji Kassim et al. 2021. Mental health nurses' views of ward readmission: A focus group study in Brunei Darussalam |
| --- |
| Findings: 3 main themes   1. the significant role of the family. (U) |
| Illustration: “I consider that family support is significant. If we think about it, the patient is unwell. If there is no such support, this can potentially lead to readmission. There are no people to monitor patient compliance. Almost all admitted patients will rely on the medication to get better? So medication compliance is critical. If we solely trust the patient to monitor their medication, I honestly think it will not be good enough. Hence, family support is essential. (Nurse 4, FGD4)” p. 404 |
| 1. the value of psychoeducation. (U) |
| Illustration: “I want to strengthen the nurse’s psychoeducation. We usually give psychoeducation to the family, particularly on the importance of medication. Unfortunately, some of them are still in denial. They may not feel that the patient needed any more medicine. Hence, they stop the medication. This patient will be soon readmitted. Therefore psychoeducation is essential for them. (Nurse 3, FGD5)” p. 405 |
| 1. Addressing the needs of patients. (U) |
| Illustration: “We are doing the job as professional nurses. We have to accept that, for any readmission, we have a job to do. It can be stressful nevertheless, mainly if the patient is reluctant to improve their health (Nurse 3, FGD5) p. 406 |

| Study 8: Hristodoulidis et al. 2022. Exploration of personal recovery-oriented care on an acute mental health unit |
| --- |
| Findings: Acute mental health nurses work within a medically dominated model of care  Collaboration with patients can decrease aggression and use of seclusion  Overall, personal recovery is difficult of acute wards  3 themes – 6 subthemes   1. a) corporeality = how the body experiences personal recovery. (U)   eg: lack of continuity of care undermines therapeutic relationship, some nurses had difficulty defining personal recovery |
| Illustration: “…how people get well and stay well … (Leigh L31) … When they [consumers] first come into acute, recovery is not always focused on (Leigh L25). p. 164 |
| b) relationality = interpersonal relationships between those experiencing personal recovery. (U) |
| Illustration: “It’s like the social workers and the well, the OTs do all the nice stuff, and we do all the nasty stuff, like call code greys and give people injections against their will (Emily L346).” p. 165 |
| c) temporality = how time is experienced in relation to personal recovery. eg: lack of time. (U) |
| Illustration: “If you’re in a one-to-one engagement with a client who might need an hour, you’ve got to hop off and do your 15-minute visuals which then ends the conversation. You’ve got to start all over again and they might feel frustrated with you having to leave and come back, leave and come back, and not engage (Fay L264-269). p. 165 |

| Study 9: Jorgensen et al. 2020. The conditions and possibilities for recovery: A critical discourse analysis in a Danish psychiatric context |
| --- |
| Interviews with nurses and patient records written by nurses were analysed with 3 dimensions of analysis – textual analysis (includes vocabulary and grammar), discursive practice and social practice  Findings: |
| 1. Textual analysis (U)    1. Vocabulary of the text   Interviews with nurses  In general nurses have an understanding that patient participation is part of recovery. Terms used e.g. “environmental therapy” “recovery oriented practice” but without direct definitions. The “recovery process” is said to be service user’s responsibility however  Illustration: “it is also hard to involve a patient who is very psychotic or does not have the energy to deal with all sorts of questions” (IW3). p. 3017  Some scepticism towards the concept of recovery  Illustration: “…they may be very bothered by hearing voices and do not have the resources to do things other than to endure the voices and they need care. The patient may lack recognition of the disorder, and it is an uphill struggle if they do not share our perception of the disease (IW 3).” p. 3017  We need to work in a recovery-oriented manner’; ‘our manager of the ward has pointed out that we need to work in a recovery-oriented manner.” “We learn it, and there is a good atmosphere in the ward to work on the recovery and participation of patients and relatives (IW 3).” p. 3017  Patient records  Notes primarily focus on the effect of medical treatment. No direct use of “recovery” as a keyword - words such as “hopes” and “service user experiences” used p. 3018. There is no information on the service user's own interpretation of his or her problems or wishes for the future.” p. 3018. A form of coded language is used:  Illustration: “action 4: he is exhausted and will not go for a walk” p.3018 (unclear if inpatient or outpatient)   - 1. Grammar of the texts   Interviews with nurses  Hierarchical and asymmetrical power relationship. “What treatment will be best depends on the problems the patient has. It’s not a free choice, but we listen a lot to the patients” (IW3). P. 3018  The nurses refer to themselves as “we”. p. 3018 and refer to the service user objectively eg: “the service user describes”. p. 3018-9  Illustration: “We try to be open to the patient’s wishes, but our structure can be a barrier. It is also we who choose the offers, methods etc. The patients have no free choice” (IW5) p. 3018  Patient records  No direct quotes from patient records |
| 1. Discourse practice (U)   Interviews with nurses.  Paternalistic and biomedical discourse  Illustration: “We cannot expect patients to make big decisions when they are psychotic etc, etc. Then we take it easy, and in my view, this isn’t a problem. If the patient is weak, we take over a little” (IW1). p. 3019  Patient records  Predominantly a biomedical deficit discourse eg: “lack of disease recognition” vocabulary such as “trauma” is rare (p. 3020) |
| 1. Social practice (US)   Recovery is considered to be open to everyone but is best suited to the most resourceful people. Nurses provide psychoeducation, considered one of the most important tasks. The biomedical discourse and governmental structures are used to interpret problems and treatment with people expected to actively comply. No illustrations/quotes are provided |

| Study 10: Lim et al. 2019. Changing practice using recovery-focused care in acute mental health settings to reduce aggression: A qualitative study |
| --- |
| Findings: 5 categories describe participants descriptions of recovery focused care and how they could be utilised to reduce aggression   1. Identify the reason for the behaviour before responding. (U) |
| Illustration: ‘Break [down] barriers [in communications] and misunderstandings of what’s going on exactly at the time. If you know they are in personal crisis or have a decompensation in mental or emotional state, being locked in a small area with fourteen or fifteen other people who are very unwell, and you are saying “no” to all their requests, I mean obviously all those things can lead to aggression as well’ (P13) pp. 240-1 |
| 1. Being sensitive to the consumers triggers for aggression. (U) |
| Illustration: “So I think the triggering factors are usually more to do with staff not being on guard to see what’s happening not giving [the person] enough attention [and] time to explain what’s going on” (P 14] p. 241 |
| 1. Focus on the consumers strengths and support, not risks. (U) |
| Illustration: “Work on a strength-based approach, assess what they [the consumers] are good at …. You could use it to collaborate with the person to find ways to resolve the problem” [P1] p. 241 |
| 1. Being attentive to the consumers needs. (U) |
| Illustration: “making the environment more conducive for them, rather than trying to control the person” (P11) |
| 1. Reconceptualize aggression as a learning opportunity. (U) |
| Illustration: “a great learning opportunity” (P11) for the consumer to improve their own ability, strengths and vulnerabilities” (P20). p. 242 |

| Study 11: McKenna et al. 2014. Recovery-oriented care in acute inpatient mental health settings: An exploratory study |
| --- |
| Findings:   1. Hope is crucial to starting the recovery journey. ( E ) |
| Illustration: “I think it’s hard in inpatient settings, often initiating recovery is not what the consumer wants. So they will come in, they will be admitted to high dependency, they will be an involuntary patient and they don’t want to be here, they don’t want to talk to you.” p. 527 |
| 1. Promoting autonomy and self-determination. (U) |
| Illustration: ““You can choose an injection or a tablet.” Like, I think sometimes we try to create choice when there is no choice. It may be an illusion of choice—“Where do you want your depot? In your arm or in your buttock?” p. 529 |
| 1. Collaborative partnerships and meaningful engagement (U) |
| Illustration: “There is so much going on. You are allocating in your mind when you are to go and spend time with that [consumer], but something else will happen.” p. 529 |
| 1. A focus on strengths. (U) |
| Illustration: “I try to identify one strength that I see in them and say, “Oh, that’s fantastic!” In the conversation I find they have a hobby or something, and I focus on that, and say how great it is that they do such and such. It also builds rapport.” p. 530 |
| 1. Holistic and personalised care. ( E ) |
| Illustration: “We talk to the family whenever they are visiting the consumers and explain what is happening. We have to maintain confidentiality according to the consumer’s permission. But if the person [consumer] is happy, we talk to the family. “ p. 530 |
| 1. Community participation and citizenship. (E ) |
| Illustration: “So our primary role as nurses . . . we are here to get them back on track, but with the time element, it’s very fast. The turnover is quick here, in-out, in-out. We do our best with the resources we’ve got, with the knowledge that we have, to empower [consumers] enough to get them going through the next step. . . . We used to take [consumers] down to the market and stuff like that. But, it has been about a year since I last did that." p. 531 |

| Study 12: Tucker et al. 2020. Recognition and management of agitation in acute mental health services: a qualitative evaluation of staff perceptions |
| --- |
| Findings: 2 themes in nurse experience of management of agitation.  1. recognition of agitation. role of the patient – self-awareness and self-report of agitation. (U)  role of the nurse – knowledge of patient’s baseline, communication, signs and symptoms of agitation |
| Illustration: “Those kind of things are obvious ones but there are much more subtle ones, if you know someone’s baseline and the way they behave when they are not agitated you start to see signs of them changing in their behavior. (Group D) p. 109 |
| 1. 2. management of agitation - types of interventions: non-pharmacological (eg verbal de-escalation) and pharmacological. (U)   – processes that support successful management – rapport, patient involvement, individualised, flexible |
| Illustration: “We go on de-escalation being the first line of call when we are trying to solve agitation on the ward” (Group C) p. 109 |

| Study 13: Waldemar et al. 2019. Recovery-oriented practice: Participant observations of the interactions between patients and health professionals in mental health inpatient settings |
| --- |
| Findings: One main theme: “as if collaborating” – shallow or artificial interactions  4 subthemes   1. Negotiating on limited grounds. (U) |
| Illustration: “The psychiatrist and nurse are sitting together preparing for a meeting … what they are going to offer her: a choice between two different medical products or electroconvulsive therapy” p 325 |
| 1. Competing demands. (U) |
| Illustration: Nurse Bina agrees with her but then says “but it is difficult, because then his relatives come and ask why we are letting him out, but we can’t just keep people locked up in here” p. 325 |
| 1. Inconsistent guidance and postponed decisions. ( E ) |
| Illustration: The nurse tells her that it’s because of a system they have so she can’t rely on having the same primary nurse every day. Sara sighs and says, “ok well, I guess I just have to deal with even more people then.” p. 325 |
| 1. Control and condescending communication. ( US ) |
| Illustration: “He then says that the patient had requested leave and adds that the patient seems quiet and calm which is why they agree on allowing him leave.” (Nurse assistant) p. 326 |

Appendix S5 : thematic analysis conducted by reviewer of included studies

Living experience perspective

| Domain number | Included studies |
| --- | --- |
| 1: Promoting a culture and language of hope and optimism |  |
| 2: Person first and holistic | Adnanes et al. 2018, Bradley et al. 2021, Eldal et al. 2019, Huang et al. 2020, Isobel et al. 2021, Muddle et al. 2024, Olasoji et al. 2018, Olasoji et al. 2020, Ould-Brahim et al. 2020, Schon 2013, Waldemar et al. 2018 |
| 3: supporting personal recovery | Bradley et al. 2021, Moreno-Poyato et al. 2021, van de Velde et al. 2021, Waldemar et al. 2018, Wilson et al. 2023, Wyder et al. 2015, Wyder et al. 2016, Wyder et al. 2018 |
| 4: organisational commitment and workforce development | Isobel et al. 2021, Moreno-Poyato et al. 2021, Muddle et al. 2024, Olasoji et al. 2018, Olasoji et al. 2020, Ould-Brahim et al. 2020, van de Velde et al. 2021, Waldemar et al. 2018, Waldemar et al. 2019, Wilson et al. 2023, Wyder et al.2015, Wyder et al. 2016, Wyder et al. 2018 |
| 5: action on social inclusion and the social determinants of health,  mental health and wellbeing | Bradley et al. 2021 |

Family perspective

| Domain number | Included studies |
| --- | --- |
| 1: Promoting a culture and language of hope and optimism |  |
| 2: Person first and holistic | Schaffer et al 2021 |
| 3: supporting personal recovery |  |
| 4: organisational commitment and workforce development | Schaffer et al 2021 |
| 5: action on social inclusion and the social determinants of health,  mental health and wellbeing |  |

Nurse perspective

| Domain number | Included articles |
| --- | --- |
| 1: Promoting a culture and language of hope and optimism |  |
| 2: Person first and holistic | Chambers et al. 2015, Cleary et al. 2013, Foster & Isobel 2018, Gwinner & Ward 2014, Haji Kassim et al. 2021, McKenna et al. 2014 |
| 3: supporting personal recovery | Chambers et al. 2015, Cheetham et al. 2018) Cleary et al. 2013, Digby et al. 2020 Gwinner & Ward 2014, Haji Kassim et al. 2021, Hristodoulidis et al. 2022, Jorgensen et al. 2020, Lim et al. 2019, McKenna et al. 2014, Tucker et al. 2020 |
| 4: organisational commitment and workforce development | Chambers et al 2015, Cleary et al. 2013, Digby et al. 2020, Foster & Isobel 2018, Gwinner & Ward 2014, Hristodoulidis et al. 2022, Jorgensen et al. 2020, Lim et al. 2019, Tucker et al. 2020, Waldemar et al. 2019 |
| 5: action on social inclusion and the social determinants of  health, mental health and wellbeing | Cleary et al. 2013, Jorgensen et al. 2020 |
